# Influence of the TARP γ8-Selective Negative Allosteric Modulator JNJ-55511118 on AMPA Receptor Gating and Channel Conductance🅂

**DOI:** 10.1124/molpharm.121.000473

**Published:** 2022-03-03

**Authors:** Ian D. Coombs, Craig A. Sexton, Stuart G. Cull-Candy, Mark Farrant

**Affiliations:** Department of Neuroscience, Physiology and Pharmacology, University College London, London, United Kingdom

## Abstract

AMPA-type gultamate receptors (AMPARs) mediate excitatory signaling in the brain and are therapeutic targets for the treatment of diverse neurological disorders. The receptors interact with a variety of auxiliary subunits, including the transmembrane AMPAR regulatory proteins (TARPs). The TARPs influence AMPAR biosynthesis and trafficking and enhance receptor responses by slowing desensitization and deactivation and increasing single-channel conductance. TARP *γ*8 has an expression pattern that is distinct from that of other TARPs, being enriched in the hippocampus. Recently, several compounds have been identified that selectivity inhibit *γ*8-containing AMPARs. One such inhibitor, JNJ-55511118, has shown considerable promise for the treatment of epilepsy. However, key details of its mechanism of action are still lacking. Here, using patch-clamp electrophysiological recording from heterologously expressed AMPARs, we show that JNJ-55511118 inhibits peak currents of *γ*8-containing AMPARs by decreasing their single-channel conductance. The drug also modifies hallmark features of AMPAR pharmacology, including the TARP-dependent actions of intracellular polyamines and the partial agonist kainate. Moreover, we find that JNJ-55511118 reduces the influence of *γ*8 on all bio-physical measures, aside from its effect on the recovery from desensitization. The drug is also effective when applied intracellularly, suggesting it may access its binding site from within the membrane. Additionally, we find that AMPARs incorporating TARP *γ*2 mutated to contain the JNJ-55511118 binding site, exhibit greater block than seen with AMPARs containing *γ*8, potentially reflecting differences in TARP stoichiometry. Taken together, our data provide new insight into the mechanism by which *γ*8-selective drugs inhibit AMPARs.

## Introduction

α-Amino-3-hydroxy-5-methyl-4-isoxazolepropionic acid (AMPA)-type glutamate receptors (AMPARs) are responsible for fast signaling and the expression of plasticity at excitatory synapses throughout the central nervous system (Hansen et al., 2021). Manipulation of AMPAR activity has been actively pursued as a possible therapy for various neurological and psychiatric disorders, including stroke, depression, pain, epilepsy, and cognitive deficit in Alzheimer’s disease (Lynch, 2006; Rogawski, 2011; Brogi et al., 2019). Although a plethora of AMPAR positive and negative allosteric modulators have been developed (Partin, 2015; Stenum-Berg et al., 2019; Frydenvang et al., 2021), these lack selectivity for different brain regions. This is because, although AMPARs formed from various combinations of the four core subunits (GluA1-4) show differential distribution, the subunits are structurally highly homologous. For example, the negative allosteric modulator perampanel has proved effective against multiple seizure types (Tsai et al., 2018; Potschka and Trinka, 2019; Hanada, 2020), but its lack of regional specificity is thought to contribute to side effects that include ataxia and dizziness (Zwart et al., 2014; Villanueva et al., 2021).

The biophysical and pharmacological properties of AMPARs depend not only on their subunit composition but also on their complement of associated proteins or auxiliary subunits (Jackson and Nicoll, 2011; Ishii et al., 2020; Coombs and Cull-Candy, 2021; Matthews et al., 2021). Of the several families of recognized AMPAR auxiliary subunits, the transmembrane AMPAR regulatory proteins (TARPs; γ2, -3, -4, -5, -7, and -8) have been the most extensively studied. Crucially, the various TARP family members are distributed differentially throughout the brain (Fukaya et al., 2005), potentially offering pharmacological targets with regional specificity (Zwart et al., 2014). Recently, a novel group of compounds were developed that selectively inhibit AMPAR complexes containing TARP *γ*8 (Gardinier et al., 2016; Kato et al., 2016; Maher et al., 2016; Ravula et al., 2018; Savall et al., 2019). Such AMPARs are enriched in neurons of the forebrain, including in hippocampal CA1 cells (Fukaya et al., 2005; Rouach et al., 2005). In preclinical studies, the *γ*8-selective blockers JNJ-55511118 (JNJ-118) and LY3130481 have shown considerable promise as treatments for epilepsy, with efficacy similar to that of perampanel but without the undesirable motor side effects (Kato et al., 2016; Maher et al., 2016).

JNJ-118 shows >1000-fold selectivity for *γ*8-containing AMPARs (Maher et al., 2016). The drug partially inhibits both peak- and steady-state glutamate–evoked currents while accelerating the kinetics of deactivation and desensitization (Maher et al., 2016; Dohrke et al., 2020). Mutagenesis, molecular modeling, and cryo-electron microscopy (cryo-EM) studies have shown that the JNJ-118 binding site lies between the third and fourth transmembrane regions (TM3 and TM4) of *γ*8 and the first membrane region (M1) of adjacent AMPAR subunit (Maher et al., 2016; Lee et al., 2017; Dohrke et al., 2020; Yu et al., 2021). Selectivity of the drug for AMPARs containing *γ*8, over those containing other TARP family members, depends on the presence of two amino acid residues within TM3 and TM4 of *γ*8. Replacing these residues abolishes JNJ-118 sensitivity, whereas introducing them into TARP *γ*2 renders AMPARs containing this mutated TARP sensitive to JNJ-118 (Maher et al., 2016).

Despite the identification of a binding pocket for JNJ-118, it remains unclear exactly how the drug diminishes the AMPAR response. Thus, although JNJ-118 inhibits the peak and steady-state glutamate–evoked current of *γ*8-associated AMPARs (Maher et al., 2016), it is not known whether this reflects a reduction in the number of functional receptors, a reduction in single-channel conductance, or a change in channel gating. Here, we examine the action of JNJ-118 on homomeric GluA2(Q) AMPARs containing *γ*8 or doubly mutated *γ*2. We show that, in addition to decreasing peak- and fractional steady-state currents and the time constants of deactivation and desensitization, JNJ-118 decreased the weighted mean single-channel conductance by reducing the proportion of high-conductance openings. JNJ-118 also increased channel block by intracellular spermine and decreased the efficacy of the partial agonist kainate but did not affect recovery from desensitization. Thus, for all but one parameter examined, JNJ-118 appears to reduce the influence of TARP on AMPAR function.

## Materials and Methods

### Heterologous Expression.

We expressed recombinant AMPAR subunits and TARPs (plus EGFP) in HEK293T/17 cells (mycoplasma-free; https://www.atcc.org). These were maintained under standard protocols, as described previously (Coombs et al., 2017). Rat GluA2 flip cDNA was unedited at the Q/R site (Q-form) and R/G edited. *γ*2 and *γ*8 cDNA were from rat. cDNA for *γ*8.DM, carrying mutations G210A and V177I that completely abolish activity of JNJ-118 (Maher et al., 2016), was from human and was a gift from Michael Maher (Janssen Research & Development L.L.C., San Diego, CA, USA). Double point mutations in *γ*2 (A184G and I153V; *γ*2.DM), corresponding to those shown to confer sensitivity to inhibition by JNJ-118 to human *γ*2 (Maher et al., 2016), were produced using standard polymerase chain reaction. AMPAR/TARP combinations were transfected at a cDNA ratio of 1:2. Transient transfection was performed using Lipofectamine 2000 (Life Technologies). Cells were split 12–30 hour after transfection and plated on glass coverslips treated with poly-L-lysine. Electrophysiological recordings were performed 18–48 hour later.

### Electrophysiology

Cells were viewed using a fixed-stage microscope (Axioskop FS1, Zeiss) and perfused at a rate of 1.5–2 ml min^–1^ with an external solution containing 145 mM NaCl, 2.5 mM KCl, 1 mM CaCl_2_, 1 mM MgCl_2_, and 10 mM HEPES, pH 7.3. Patch-clamp electrodes were pulled from borosilicate glass (1.5 mm o.d., 0.86 mm i.d.; Harvard Apparatus) and fire polished to a final resistance of 8–12 MV. The internal solution contained 145 mM CsCl, 2.5 mM NaCl, 1 mM Cs-EGTA, 4 mM MgATP, and 10 mM HEPES (pH 7.3 with CsOH) supplemented with 100 mM spermine tetrahydrochloride (Tocris Bioscience). Recordings were made from outside-out patches at 22–25°C using an Axopatch 200B amplifier (Molecular Devices). Currents were recorded at −60 mV, low-pass filtered at 10 kHz, and digitized at 20 kHz using an NI USB-6341 (National Instruments) interface with Strathclyde Electrophysiology Software WINWCP (John Dempster, University of Strathclyde, Glasgow, UK).

### Rapid Agonist Application to Excised Patches

Rapid agonist application was achieved by switching between continuously flowing solutions. Solution exchange was achieved by moving an application tool made from theta glass (Hilgenberg) or triple-barreled glass (Vitrocom) mounted on a piezoelectric translator (Physik Instrumente). JNJ-55511118 (Tocris) was used at the indicated concentrations. The 10%–90% exchange time, assessed by jumping open electrodes into a diluted solution and observing junction potential changes, were between 120–300 μs.

### Data Analysis

Records were analyzed using Igor Pro 6.35 (Wave-metrics) with Neuromatic 2.8 (http://www.neuromatic.thinkrandom.com/). Entry into desensitization (200 millisecond application of 10 mM glutamate) and current deactivation (1 to 2 millisecond application of 10 mM glutamate) were fitted with the sum of two exponentials and the weighted time constants (τ_w, des_ and τ_w, deact_) calculated, according to:

(1) $$\tau_{\text{w}}=\tau_{\text{f}}\left(\frac{A_{\text{f}}}{A_{\text{f}}+A_{\text{s}}}\right)+\tau_{\text{s}}\left(\frac{A_{\text{s}}}{A_{\text{f}}+A_{\text{s}}}\right)$$

where *A*_f_ and τ_f_ are the amplitude and time constant of the fast component, and *A*_s_ and τ_s_ are the amplitude and time constant of the slow component.

Nonstationary fluctuation analysis was performed on the decaying phase of currents evoked by 1 or 200 millisecond applications of 10 mM glutamate (30–200 successive applications), as previously described (Soto et al., 2007). The variance for each successive pair of current responses was calculated and the single-channel current (*i*) and total number of channels (*N*) were then determined by plotting the ensemble variance (*σ*^2^) against mean current (*Ī*) and fitting with a parabolic function:

(2) $$\sigma^{2}=i\bar{I}-{\bar{I}}^{2}/N+\sigma_{\text{B}}^{2}$$

where σ_B_^2^ is the background variance. The weighted mean single-channel conductance was calculated from the single-channel current and the holding potential.

Records used for single-channel analysis were digitally filtered at 4 kHz and individual channel events were selected by eye. Channel openings were analyzed using QuB (ver. 2.0.0.20; https://qub.mandelics.com). The amplitude of the resolved openings was measured from either the entire opening when they occurred during steady-state or from closing transitions alone (final current level to adjacent base-line) when part of the initial decay. Ambiguous events were excluded from analysis. Measured openings (at –60 mV) were binned by current and fitted using a multipeak Gaussian function (IGOR Pro).

Rectification index (RI) (*I*_+60_/*I*_–60_) was calculated as the ratio of peak currents at +60 mV/–60 mV. *G-V* relationships were calculated from peak currents measured at 10 mV intervals between –110 and +80 mV. TARP-free GluA2 currents displayed minimal outward rectification, and *G-V* curves were fitted with the Boltzmann equation:

(3) $$G=G_{\max}\left(\frac{1}{1+\exp\left(\frac{V_{\text{m}}-V_{\text{b}}}{k_{\text{b}}}\right)}\right)$$

where *G*_max_ is the conductance at a sufficiently hyperpolarized potential to produce full relief of polyamine block, *V*_m_ is the membrane potential, *V*_b_ is the potential at which 50% of block occurs, and *k*_b_ is a slope factor describing the voltage dependence of block (the membrane potential shift necessary to cause an e-fold change in conductance). GluA2 coexpressed with TARPs displayed double rectification necessitating *G-V* curves be fitted with a double Boltzmann equation containing equivalent terms for voltage-dependent permeation (p) (Panchenko et al., 1999):

(4) $$G=G_{\max}\left(\frac{1}{1+\exp\left(\frac{V_{\text{m}}-V_{\text{b}}}{k_{\text{b}}}\right)}\right)+G_{\max,\text{p}}\left(\frac{1}{1+\exp\left(\frac{V_{\text{m}}-V_{\text{p}}}{k_{\text{p}}}\right)}\right)$$

*V*_b_ values from both Boltzmann equations were compared between conditions.

Recovery from steady-state desensitization was measured following a 300-millisecond equilibrating application of 10 mM glutamate. The recovery of glutamate-activated peak currents was measured following 2–500 millisecond intervals in control solution and fit with a single exponential to obtain the time constant of recovery (*τ*_rec_). The kainate/glutamate ratio (*I*_KA_/*I*_Glu_) was measured by dividing the current produced by 50 μM kainate by that produced by 1 mM glutamate in the continuous presence of 50 μM cyclothiazide.

### Data Presentation and Statistical Analysis

Summary data are presented in the text and in Tables 1–3 as mean ± S.D. from n patches together with paired or unpaired mean differences with 95% confidence intervals (lower bound, upper bound) and *P* values from one-or two-sample tests. Statistical tests were performed using R (version 4.1.1, the R Foundation for Statistical Computing, https://www.r-project.org/) and R Studio (version 1.4.1717, RStudio). Bias-corrected and accelerated confidence intervals were calculated from 5000 bootstrap resamples using the dabestr package (Ho et al., 2019). Normality was not tested statistically but gauged from density histograms and/or quantile-quantile plots. On the basis of this, non-parametric tests were used throughout. Although illustrated separately in the figures, for each measure, a statistical comparison was performed across the five different receptor types (GluA2, GluA2/*γ*8, GluA2/*γ*8.DM, GluA2/*γ*2, and GluA2/*γ*2.DM) as a single combined analysis. In the case of *I*_KA_/*I*_glu_, only GluA2, GluA2/*γ*8 and GluA2/*γ*2. DM were examined, thus the statistical comparison was performed across three different receptor types. Omnibus tests were performed using Kruskal-Wallis rank-sum test or rank-based longitudinal (repeated measures) analysis using the nparLD package (Noguchi et al., 2012). One- or two-sample tests were performed using one-sample Wilcoxon signed-rank tests (against 100%), Wilcoxon signed-rank tests for paired comparisons, or Wilcoxon rank-sum tests for unpaired comparisons. For pairwise tests, calculated *P* values were adjusted for multiple comparisons within each separate family of comparisons using Holm’s sequential Bonferroni correction (mt.rawp2adjp function in the R package multtest; Pollard et al., 2005). The results of the different families of statistical tests (one for each measure) are presented in Tables 1–3 and Supplemental Table 1. No statistical test was used to predetermine sample sizes; these were based on standards of the field. No randomization was used.

## Results

### JNJ-118 Decreases Peak Current and Modifies Kinetics of GluA2(Q)/*γ*8

**Fig. 1:**
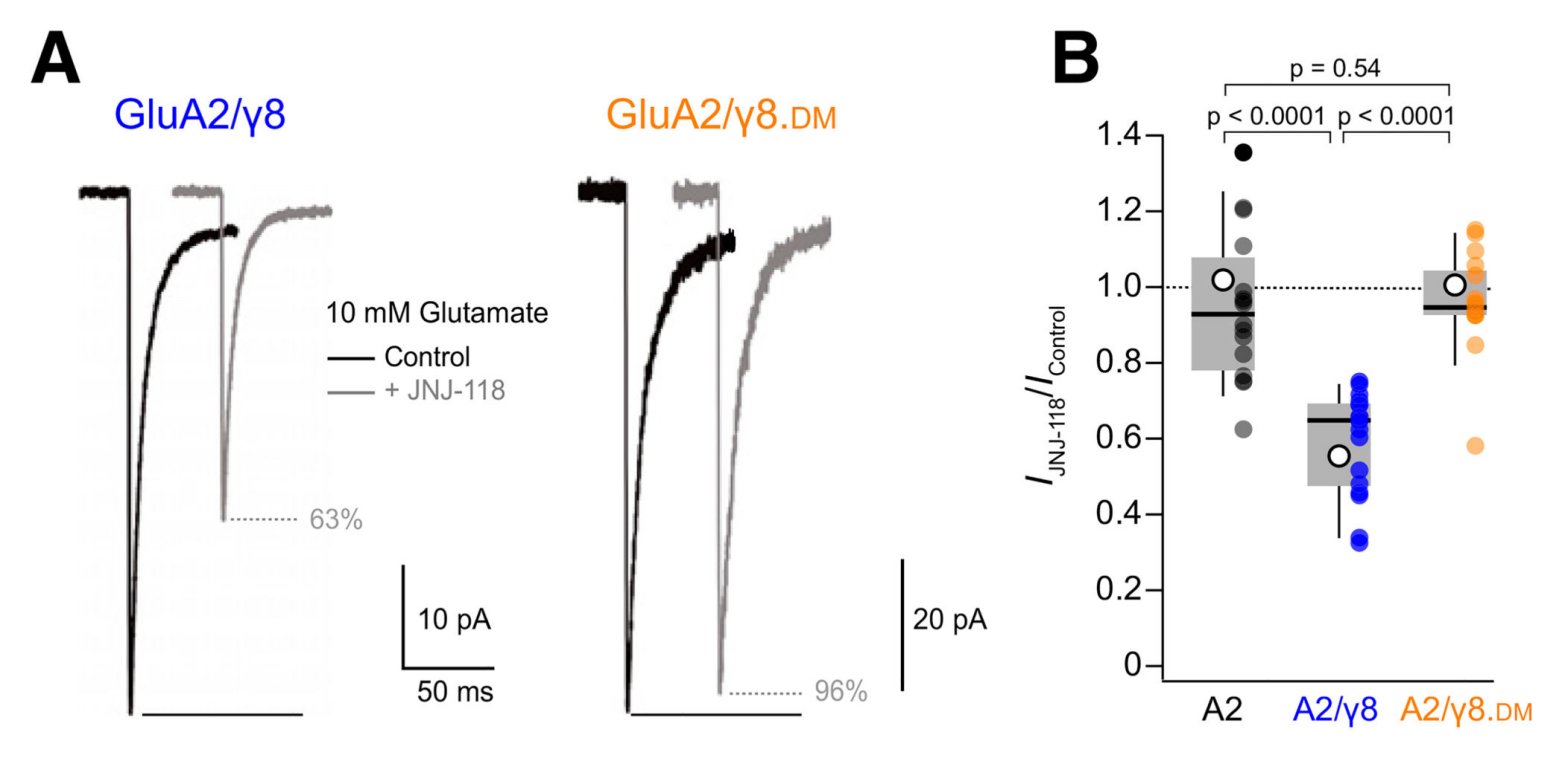
JNJ-118 decreases peak amplitude of currents from GluA2(Q)/*γ*8. (A) Representative outside-out patch responses (10 mM glutamate, 200 milliseconds, –60 mV) from two HEK293 cells transfected with GluA2/*γ*8 (left) or GluA2/*γ*8.DM (right) in control conditions (black) and in the presence of 1 μM JNJ-118 in both control and glutamate solutions (gray). Only the initial part of each response is shown, with the percent peak current remaining in JNJ-118 indicated. (B) Pooled peak inhibition data (*I*_JNJ-118_/*I*_Control_) showing the effect of 1 μM JNJ-118 on GluA2 alone, GluA2/*γ*8, and GluA2/*γ*8.DM. Box-and-whisker plots indicate the median (black line), the 25th–75th percentiles (box), and the 10th–90th percentiles (whiskers); filled circles are data from individual patches, and open circles indicate means. Indicated *P* values (adjusted for multiple comparisons as described in Table 1) are from two-sided Wilcoxon rank-sum tests following a nonparametric omnibus test (Supplemental Table 1).

We initially examined the actions of JNJ-118 on responses evoked by fast application of glutamate (10 mM, 200 millisecond, −60 mV) onto outside-out patches from HEK293T/17 cells expressing GluA2(Q) in the absence or presence of TARP *γ*8. As expected, 1 μM JNJ-118 had no effect on glutamate-evoked peak currents in the absence of *γ*8 but substantially reduced these (by ~40%) in the presence of *γ*8 (Fig. 1, A and B; Table 1). In cells transfected with GluA2 and a mutated *γ*8 (*γ*8.DM) lacking the two amino acid residues previously shown to be critical in forming the JNJ-118 binding site (Maher et al., 2016; *γ*8.DM), THE EFFECT OF JNJ-118 WAS LOST (FIG. 1, A AND B; Table 1).

Both *γ*8 and *γ*8.DM increased the weighted mean time constant of desensitization (*τ*_w, des_) and the fractional steady-state component (*I*_ss_/*I*_peak_) seen with 200 millisecond glutamate applications, as well as the weighted mean time constant of deactivation (*τ*_w, deact_) following 1- to 2-millisecond glutamate applications (Table 2). These observations confirm the incorporation of the TARPs into functional AMPARs (Cho et al., 2007). In accord with the TARP-dependent effects on peak amplitude, μM JNJ-118 decreased *τ*_w, des_, *I*_ss_/*I*_peak_ and *τ*_w, deact_ of GluA2/*γ*8 but had no effect on these measures from GluA2/*γ*8.DM (Table 2; Supplemental Table 1; Supplemental Fig. 1).

Overall, the effects of JNJ-118 on peak current, fractional steady-state current, and deactivation and desensitization of GluA2/*γ*8 were qualitatively comparable to those originally observed with GluA1/*γ*8 receptors (Maher et al., 2016). Of note, although the effects of JNJ-118 we observed were marked, in the presence of the drug, the values of *τ*_w, deact_, *τ*_w, des_ and *I*_ss_/*I*_peak_ remained different from those seen with GluA2 alone (Table 2; *τ*_w, deact_ unpaired mean difference GluA2/*γ*8/JNJ-118 minus GluA2/JNJ-118 0.48 millisecond [0.23, 0.86], *P* =0.026; *τ*_w, des_ 2.79 millisecond [1.84, 4.01], *P* < 0.0001; *I*_ss_/*I*_peak_ 1.84% [1.03, 2.76], *P =* 0.0095), suggesting that the drug does not simply eliminate these functional effects of *γ*8.

### The Proportion of Higher-Conductance Channel Openings Is Reduced by JNJ-118

**Fig. 2:**
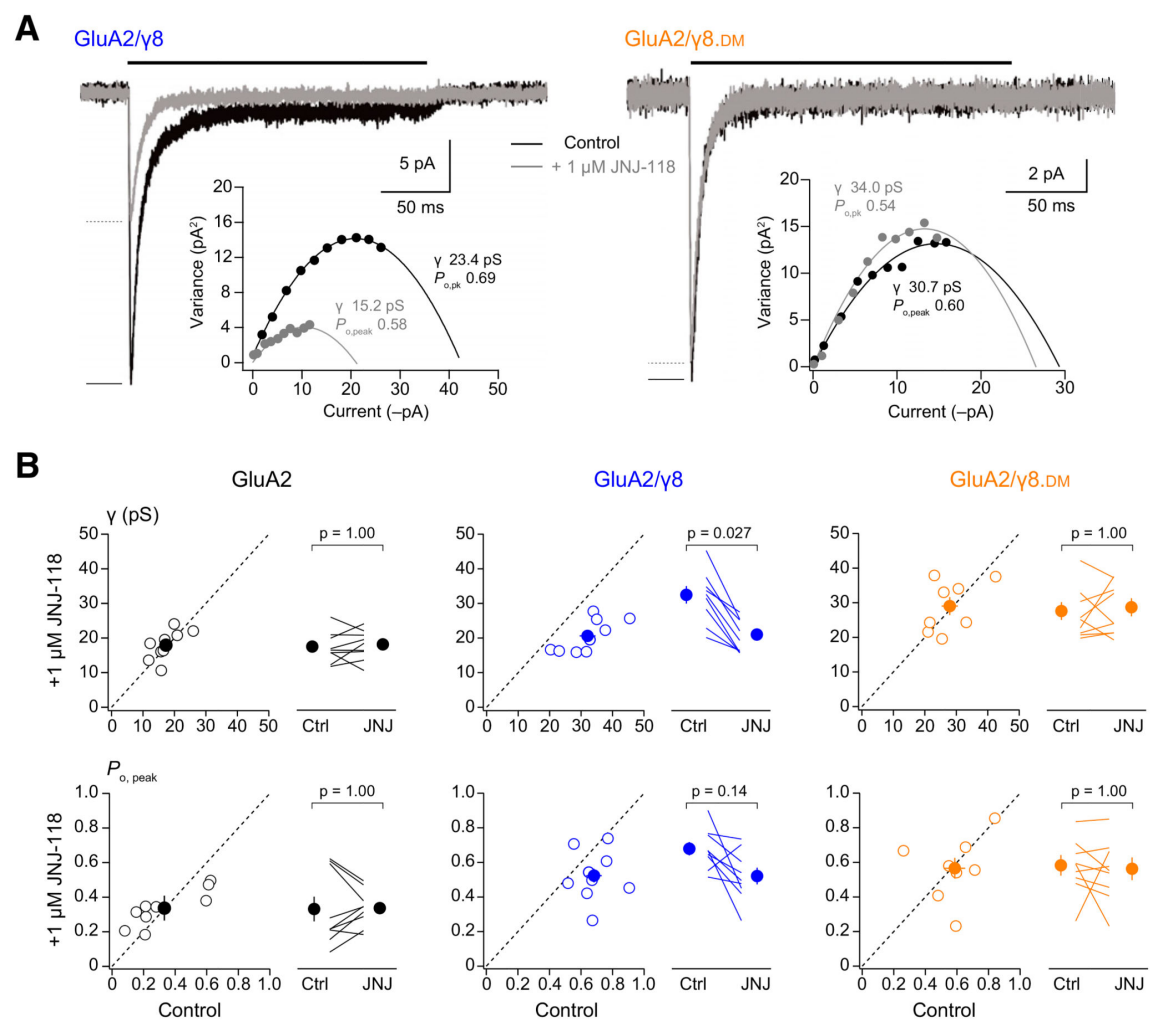
JNJ-118 decreases the weighted mean channel conductance of GluA2(Q)/*γ*8. (A) Representative outside-out patch responses (10 mM glutamate, 200 milliseconds) (black bars) recorded at –60 mV from HEK293 cells transfected with GluA2/*γ*8 (left) or GluA2/*γ*8.DM (right) in control conditions (black) or in the presence of 1 μM JNJ-118 (gray). Insets show corresponding current-variance relationships and estimated channel conductance (*γ*) and peak open probability (*P*_o, peak_). (B) Scatter and paired plots showing the effects of 1 μM JNJ-118 on weighted mean channel conductance (*γ*) and *P*_o, peak_ values for GluA2, GluA2/*γ*8, and GluA2/*γ*8.DM. Open circles show individual values, and filled circles denote the means, with error bars indicating S.E.M. In scatter plots, dashed lines denote equality, with points below the lines indicating inhibitory effects of JNJ-118. Indicated *P* values (adjusted for multiple comparisons as described in Table 1) are from two-sided Wilcoxon signed-rank exact tests following a nonparametric omnibus test (Supplemental Table 1).

Although the effect of JNJ-118 on desensitization is consistent with its effect on the steady-state current, it cannot easily account for the decrease in peak current. Indeed, an inhibitory effect of JNJ-118 on peak response persists when desensitization is blocked by cyclothiazide (Maher et al., 2016). However, as TARPs are known to increase AMPAR channel conductance – either by increasing the prevalence of high conductance openings or by increasing the absolute conductance (Tomita et al., 2005; Shelley et al., 2012) – a reduction in this effect could account for the inhibition of peak current (Maher et al., 2016). To investigate this, we used nonstationary fluctuation analysis (NSFA), an approach we have previously shown to capture the increased weighted mean channel conductance caused by TARP-association (Soto et al., 2007; Soto et al., 2009; Coombs et al., 2012). NSFA (Fig. 2A) revealed that coassembly with wild-type or mutated *γ*8 increased the weighted mean single-channel conductance and peak open probability (*P*_o, peak_) of GluA2 (Fig. 2B; Table 3). As seen with peak current, *τ*_w, des_ and *I*_ss_/*I*_peak_, JNJ-118 (1 μM) decreased both the weighted mean conductance and *P*_o, peak_ of GluA2/*γ*8 but not of GluA2/*γ*8.DM (Fig. 2B; Table 3).

**Fig. 3:**
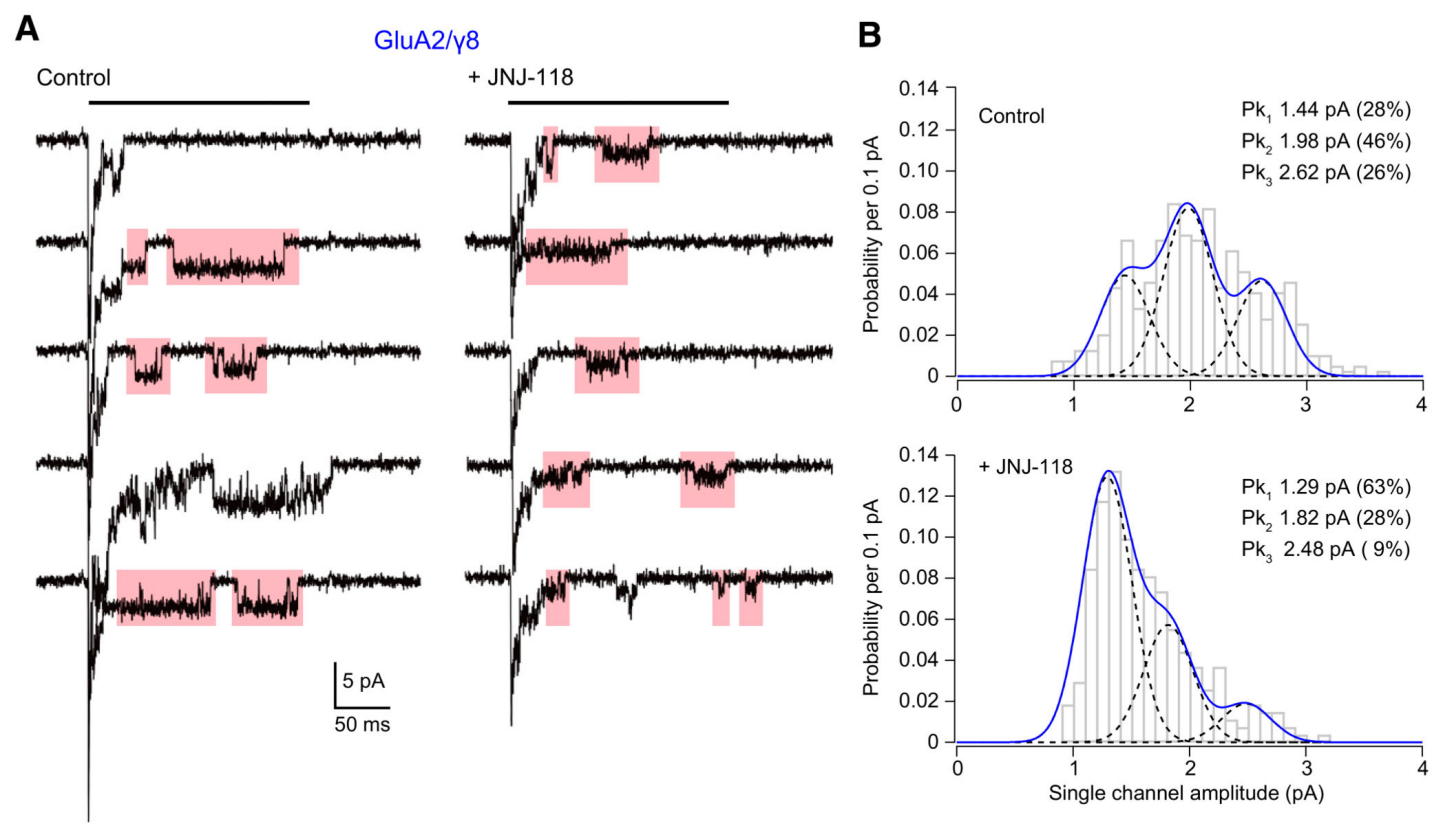
JNJ-118 reduces the prevalence of higher conductance openings of GluA2(Q)/*γ*8 channels. (A) Representative responses (10 mM glutamate, 200 milliseconds; black bars) recorded at –60 mV from an outside-out patch expressing GluA2/*γ*8 in the presence and absence of 1 μM JNJ-118. Five consecutive sweeps are shown in each condition; the initial peak is truncated, and the single-channel openings from these sweeps that were included in the analysis are highlighted. Note the prevalence of lower amplitude events in the presence of JNJ-118. (B) Pooled amplitude histograms of resolved GluA2/*γ*8 openings in the absence (top) and presence (bottom) of JNJ-118 (392 and 272 openings from 8 and 6 patches, respectively). Note the skew in amplitudes toward lower values in the presence of JNJ-118. Dotted black lines are individual gaussian fits with indicated means and proportions. Solid blue lines are the sums of the fitted gaussians.

To establish whether the reduction in mean channel conductance produced by JNJ-118 arose from a uniform or differential effect on subconductance levels, we next examined individual channel openings from outside-out membrane patches that contained only a small number of receptors. Glutamate (10 mM) was applied for 200 millisecond (Fig. 3A) and channel amplitudes measured from well-resolved openings (see Methods). Both in the absence and presence of JNJ-118, the histogram of channel amplitudes (pooled from 6 and 7 patches, respectively) could be fitted with three Gaussian components, identifying three main conductance states of approximately 23, 32, and 43 picosiemens (pS) (Fig. 3B). Although the absolute positions of these peaks were unaffected by JNJ-118, the relative prevalence of the lowest conductance was increased (from 28% to 63%) (Fig. 3B). These data suggest that the effect of JNJ-118 can be ascribed to a reduction in the proportion of the higher conductance openings rather than a decrease in the mean amplitude of all sublevels.

### JNJ-118 Reduces the Effect of *γ*8 on GluA2(Q) Spermine Block

**Fig. 4:**
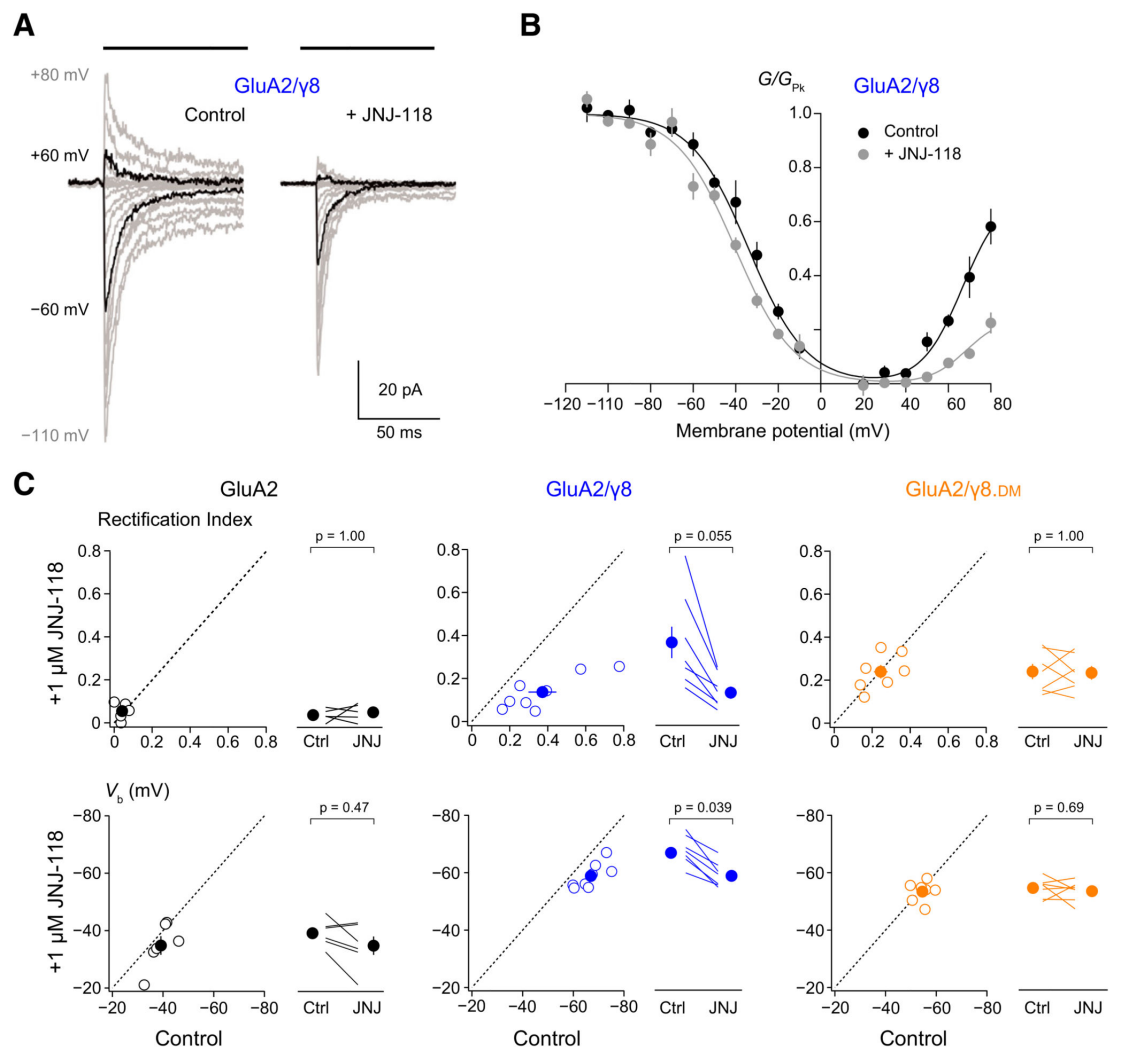
JNJ-118 increases spermine block of GluA2(Q)/*γ*8 receptors. (A) Representative responses evoked by 10 mM glutamate (200 milliseconds; black bars) recorded at potentials between –110 mV and +80 mV from an outside-out patch in the absence (left) and presence (right) of 1 μM JNJ-118. In each case, the responses at –60 and +60 mV (from which RI was calculated) are shown in black. (B) Pooled normalized conductance-voltage relationships for GluA2/*γ*8 in the absence and presence of 1 μM JNJ-118. The filled symbols are the mean values from 6 cells (with error bars showing S.E.M.), and the solid lines are fits of double Boltzmann relationships (see Methods). (C) Scatter and paired plots (as in Fig. 2) showing the effects of 1 μM JNJ-118 on Rectification Index and *V*_b_ (from individual double Boltzmann fitted conductance-voltage relationships) for GluA2, GluA2/*γ*8, and GluA2/*γ*8.DM. The indicated *P* values (adjusted for multiple comparisons as described in Table 1) are from two-sided Wilcoxon signed-rank exact tests following a nonparametric omnibus test (Supplemental Table 1).

TARP coassembly with AMPARs has previously been shown to attenuate channel block of GluA2(R)-lacking calcium-permeable AMPARs by endogenous intracellular polyamines (Cho et al., 2007; Soto et al., 2007; Soto et al., 2009; Brown et al., 2018; Coombs et al., 2021). As our data on channel conductance and kinetics indicate that the effects of JNJ-118 correspond to a partial masking of the influence of *γ*8, and polyamine block is influenced by ion flux (Bowie et al., 1998), we next asked whether TARP attenuation of polyamine block was similarly affected by the drug. Thus, we examined the effect of JNJ-118 on the voltage dependence of GluA2, GluA2/*γ*8, and GluA2/*γ*8.DM current amplitude in the presence of intracellular spermine (100 μM) (Fig. 4A). The rectification index (RI; *I*_+60_/*I*_–60_) was increased when GluA2 was coexpressed with either *γ*8 or *γ*8.DM (Fig. 4B), consistent with the view that TARP incorporation decreases spermine block. Application of 1 μM JNJ-118 decreased the RI for GluA2/*γ*8 but not that for GluA2 expressed alone or coexpressed with *γ*8.DM (Table 3).

To probe further the effect of JNJ-118 on spermine block, we generated conductance-voltage (*G*/*V*) relationships for the different receptor/TARP combinations (Fig. 4C). This revealed a drug-induced depolarizing shift in *V*_b_ (voltage giving 50% block in the negative limb of the double Boltzmann fit) for GluA2/*γ*8 but not for GluA2 alone nor for GluA2/*γ*8.DM (Table 3). This is consistent with the view that spermine block (on GluA2/*γ*8) is increased in the presence of JNJ-118. However, it is of note that in the presence JNJ-118, the *V*_b_ value for GluA2/*γ*8 did not return to its TARP-free value (Fig. 4C; Table 3; *V*_b_ unpaired mean difference GluA2/*γ*8/JNJ-118 minus GluA2/JNJ-118 24.1 mV [18.7, 31.9] *P* = 0.0087).

### Lack of Effect of Channel-Gating State on JNJ-118 Inhibition

**Fig. 5:**
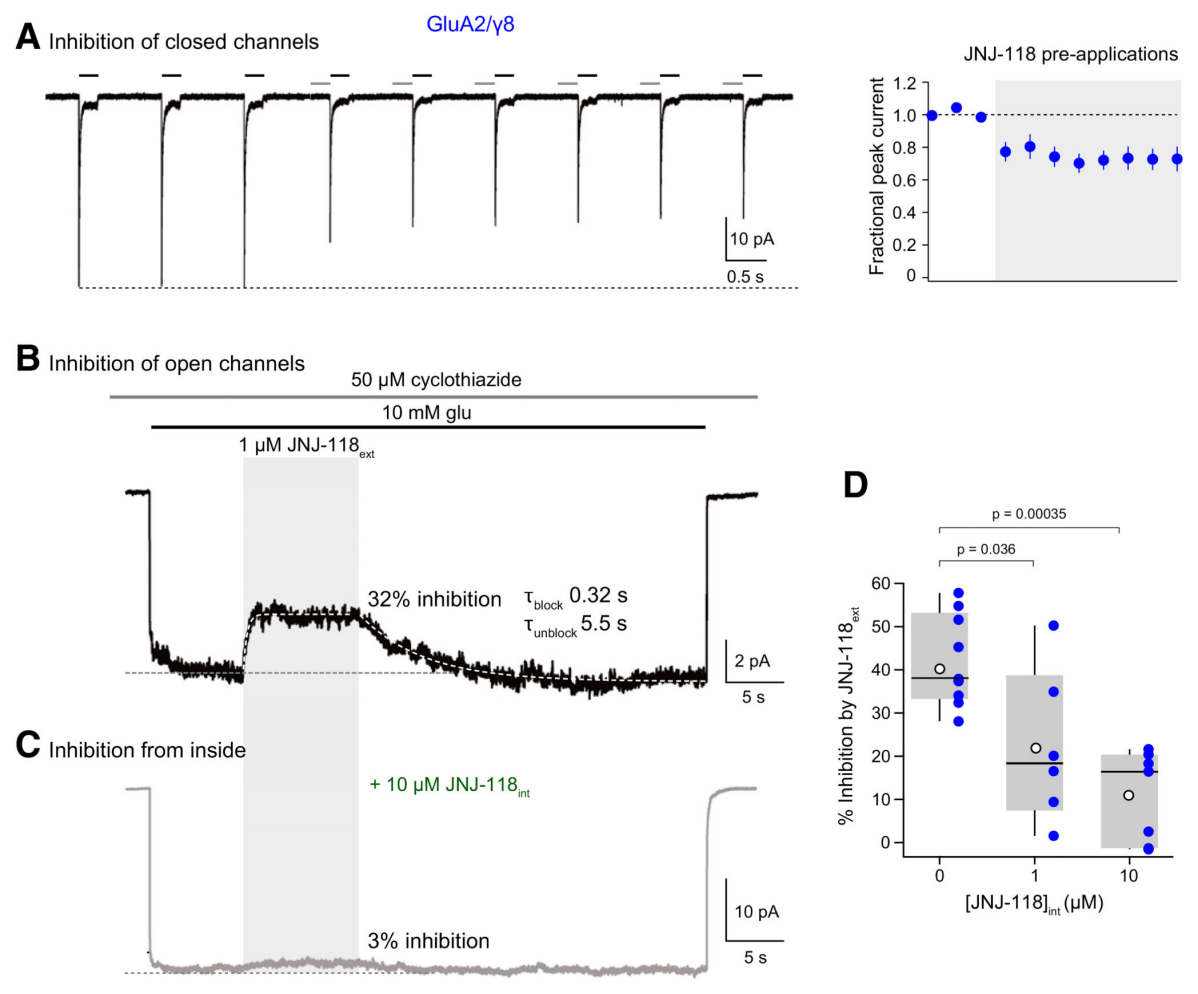
Inhibition by JNJ-118 is unaffected by channel state, and the drug is effective when applied intracellularly. (A) Representative concatenated responses from a GluA2/*γ*8 outside-out patch evoked by applications of 10 mM glutamate (200 milliseconds, 1 Hz; black bars) at –60 mV, showing inhibition produced by preapplications of 1 μM JNJ-118 (200 milliseconds; gray bars). Right-hand panel shows mean peak current data from 7 records (error bars indicate S.E.M.), normalized in each case to the mean of three applications delivered before the first preapplication of JNJ-118. (B) Representative response from a GluA2/*γ*8 outside-out patch produced by a 48-second application of 10 mM glutamate (black bar) in the constant presence of 50 μM cyclothizide. Filled gray area denotes the rapid application of 1 μM JNJ-118 for 10 seconds. White dotted lines are single exponential fits showing the timecourse of block and unblock. (C) Representative response, as in panel B, but recorded with an internal solution containing 10 μM JNJ-118. Note that in this case, the extracellular application of JNJ-118 produced a greatly reduced block. (D) Pooled data showing the degree of inhibition produced by 1 μM JNJ-118_ext_ when the internal solution contained either 0, 1, or 10 μM JNJ-118. Box-and-whisker plots as in Fig. 1. Indicated *P* values (adjusted for multiple comparisons using Holm’s sequential Bonferroni correction) are from two-sided Wilcoxon rank-sum tests following Kruskal-Wallis rank-sum test (Supplemental Table 1).

It has been suggested that binding of *γ*8-selective negative allosteric modulators to TM3 and TM4 of *γ*8 may hamper AMPAR channel opening by interfering with M3 motion, restricting expansion of the M3 gating helices (Lee et al., 2017; Yu et al., 2021). If this is indeed the case, it seems possible that the JNJ-118 binding site could be occluded by agonist-induced movement of the M3 domain. Thus, we next sought to determine whether the drug produced similar inhibition when applied to receptors with open or closed channels. We first examined JNJ-118 inhibition by applying 1 μM JNJ-118 for 200 milliseconds immediately prior to the fast application of glutamate in the absence of JNJ-118 (Fig. 5A; see Methods). A single application of JNJ-118 to receptors with closed channels was sufficient to cause 23 ± 15.9% (mean ± S.D., n = 7) inhibition of the subsequent glutamate response. Following several applications, the extent of inhibition stabilized at ~30% (Fig. 5A).

We next asked whether JNJ-118 was effective against receptors with open channels. These experiments were performed in the presence of the positive allosteric modulator cyclothiazide (50 μM) to suppress AMPAR desensitization. We were mindful that AMPARs containing *γ*8 can slowly transition into high conductance and high open probability states following activation. This process, termed superactivation (Carbone and Plested, 2016) or resensitization (Kato et al., 2010), is seen as a slow “run-up” in current and is particularly evident in the presence of cyclothiazide (Carbone and Plested, 2016; Riva et al., 2017). To allow for the development of superactivation, we used an 8-second preconditioning application of glutamate and cyclothiazide before applying JNJ-118. Following activation, as expected, GluA2/*γ*8 receptors displayed a slow run-up (Fig. 5B; 23.7 ± 18.8%, n = 7) qualitatively similar to that previously reported (Riva et al., 2017). After a rapid switch to a glutamate/cyclothiazide solution containing 1 μM JNJ-118, currents were inhibited by 39.9 ± 7.7%. The block proceeded with a weighted time constant (*τ*_block_) of 471 ± 225 millisecond, whereas on removal of JNJ-118, unbinding was slow (*τ*_unblock_ 11.8 ± 8.2 second). Given the extremely high open probability expected for superactive GluA2/*γ*8 receptors in the presence of cyclothiazide (Carrillo et al., 2019), these receptors would be closed for only a small fraction of the time. Despite this, the onset of block of the open receptors was qualitatively similar to the kinetics of block of closed receptors (Fig. 5A). Taken together, these observations suggest that the JNJ-118 binding site is not occluded by channel opening.

### JNJ-118 is Effective When Present in the Intracellular Medium

Binding of *γ*8-selective negative allosteric modulators occurs within the transmembrane region of the AMPAR complex (Kato et al., 2016; Maher et al., 2016; Dohrke et al., 2020; Yu et al., 2021). Although access from the extracellular milieu has been postulated for LY3130481/CERC-611 (Lee et al., 2017; Dohrke et al., 2020), we wondered whether JNJ-118 could access its binding site from within the lipid bilayer. If this were the case, JNJ-118 might be expected to be effective when included in the intracellular recording solution. To test this, we supplemented the pipette solution with 1 or 10 μM JNJ-118 (JNJ-118_int_) and examined whether glutamate-evoked currents remained sensitive to extracellular applications of the drug (1 μM JNJ-118_ext_) (Fig. 5C). We found that the JNJ-118_ext_–sensitive component of glutamate/cyclothiazide currents was reduced by JNJ-118_int_. Specifically, 1 μM JNJ-118_int_ reduced the inhibition caused by 1 μM JNJ-118_ext_ from ~40% inhibition in control (Fig. 5B) to 21.8 ± 16.2% (n = 6), whereas 10 μM JNJ-118_int_ reduced inhibition further to just 10.9 ± 10.6% (n = 7) (Fig. 5D). Thus, JNJ-118 appears able to access its binding site from the lipid bilayer. Although it is formally possible that JNJ-118 may bind at an additional “intracellular” site that occludes, via allostery, its action from the outside, in structural studies such binding has not been observed (Yu et al., 2021).

### Functional Effects of Incorporating a JNJ-118 Binding Site into TARP *γ*2

**Fig. 6:**
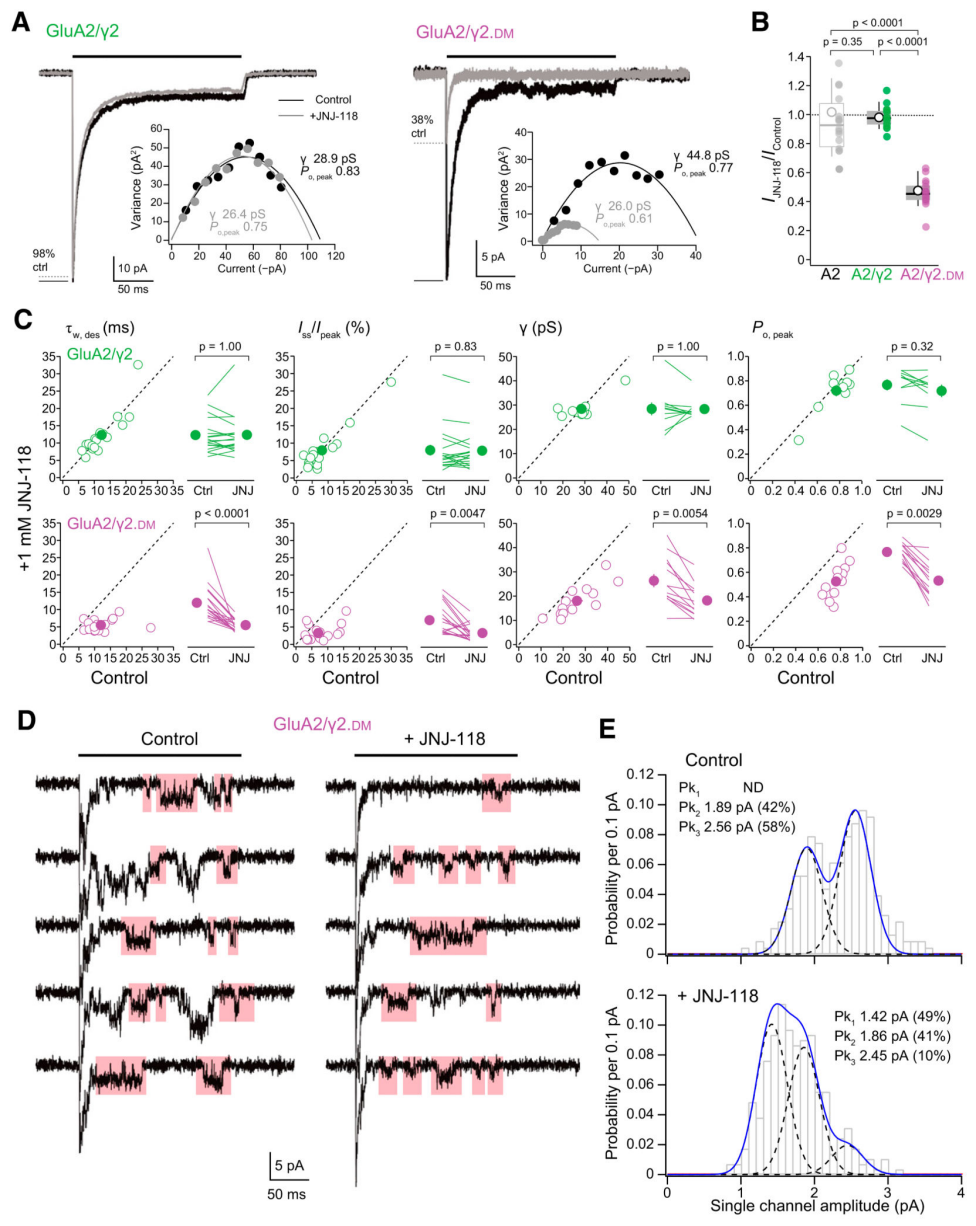
A double point mutation in TARP *γ*2 introduces JNJ-118 sensitivity to GluA2(Q)/*γ*2. (A) Representative outside-out patch responses (10 mM glutamate, 200 milliseconds) (black bars) recorded at –60 mV from HEK293 cells transfected with GluA2/*γ*2 (left) or GluA2/*γ*2.DM (right) in control conditions (black) or in the presence of 1 μM JNJ-118 (gray). Insets show corresponding current-variance relationships and estimated channel conductance (*γ*) and peak open probability (*P*_o, peak_). (B) Pooled peak inhibition data (*I*_JNJ-118_/*I*_Control_) showing the effect of 1 μM JNJ-118 on GluA2 alone (from Fig. 1B), GluA2/*γ*2, and GluA2/*γ*2.DM. Box-and-whisker plots as in Fig. 1. Indicated *P* values are from two-sided Wilcoxon rank-sum tests (adjusted for multiple comparisons as described in Table 1) following a nonparametric omnibus test (Supplemental Table 1). (C) Scatter and paired plots (as in Fig. 2) showing the effects of 1 μM JNJ-118 on the weighted mean time constant of desensitization (τ_w, des_), the fractional steady-state component (*I*_ss_/*I*_peak_), the weighted mean channel conductance, and *P*_o, peak_ values for GluA2/*γ*2 and GluA2/*γ*2.DM. Indicated *P* values are from two-sided Wilcoxon signed-rank exact tests (adjusted as described in Table 1) following a nonparametric omnibus test (Supplemental Table 1). (D) Representative responses (10 mM glutamate, 200 milliseconds; black bars) recorded at –60 mV from an outside-out patch expressing GluA2/*γ*2.DM in the presence and absence of 1 μM JNJ-118. Five consecutive sweeps are shown in each condition; the initial peak is truncated, and selected single-channel openings are highlighted. Note the prevalence of lower amplitude events in the presence of JNJ-118. e) Pooled amplitude histograms of resolved GluA2/*γ*2.DM openings in the absence (top) and presence (bottom) of JNJ-118 (228 and 289 openings from 2 and 3 patches, respectively). Note the skew in amplitudes toward lower values in the presence of JNJ-118. Dotted black lines are individual gaussian fits with indicated means and proportions. Solid blue lines are the sums of the fitted gaussians.

Although the action of JNJ-118 is *γ*8-selective, a JNJ-118 binding site can be incorporated into TARP *γ*2 by introducing mutations that are the inverse of those that remove the binding site from *γ*8 (Maher et al., 2016). Thus, receptors containing doubly mutated *γ*2 (*γ*2.DM) were previously shown, in a whole-cell Ca^2+^ influx assay, to be sensitive to JNJ-118 (Maher et al., 2016). However, details of the block were not described. To address this, we compared the effects of JNJ-118 on currents produced by fast application of glutamate onto GluA2/*γ*2 and GluA2/*γ*2.DM (Fig. 6A; Supplemental Fig. 2A).

As expected, JNJ-118 had no effect on the peak current, fractional steady-state current, or desensitization or deactivation kinetics of wildtype GluA2/*γ*2. By contrast, it decreased the peak (Fig. 6B) and fractional steady-state currents while accelerating the desensitization and deactivation kinetics of GluA2/*γ*2.DM (Tables 1 and 2; Supplemental Fig. 2A). Interestingly, with GluA2/*γ*2.DM, the inhibition of peak amplitude by JNJ-118 was somewhat greater than that seen with GluA2/*γ*8 (Table 1) (unpaired mean difference GluA2/*γ*8 minus GluA2/*γ*2.DM −13.7% [−19.9, −6.4], *P* =0.0021). Examination of the voltage dependence of currents in the presence of intracellular spermine (Supplemental Fig. 2B) showed that when GluA2 was coexpressed with either *γ*2 or *γ*2.DM, RI was increased and *V*_b_ shifted to more depolarized values (Table 3). Although application of 1 μM JNJ-118 affected neither measure for GluA2/*γ*2, it decreased RI and caused a hyperpolarizing shift in *V*_b_ of GluA2/*γ*2.DM (Supplemental Fig. 2B; Table 3).

As with *γ*8-containing receptors, we next examined the effect of JNJ-118 on channel conductance of *γ*2-containing receptors. NSFA indicated that JNJ-118 decreased the weighted mean single-channel conductance and peak open probability of GluA2/*γ*2.DM but not of GluA2/*γ*2 (Fig. 6C; Table 3). Again, in patches containing few channels, we resolved the single-channel openings during the steady-state period that followed the initial peak current. Looking jointly at channels resolved in two control and three JNJ-118–treated patches revealed the presence of three conductance levels (with means of approximately 24, 31, and 42 pS). These states contributed 0%, 42%, and 58% of openings in control conditions, compared with 49%, 41%, and 10% in the presence of JNJ-118 (Fig. 6D). Hence, as with *γ*8-associated receptors, JNJ-118 increased the proportion of lower-conductance openings arising from GluA2/*γ*2.DM.

### JNJ-118 Influences Agonist Efficacy but Not Recovery from Desensitization

**Fig. 7:**
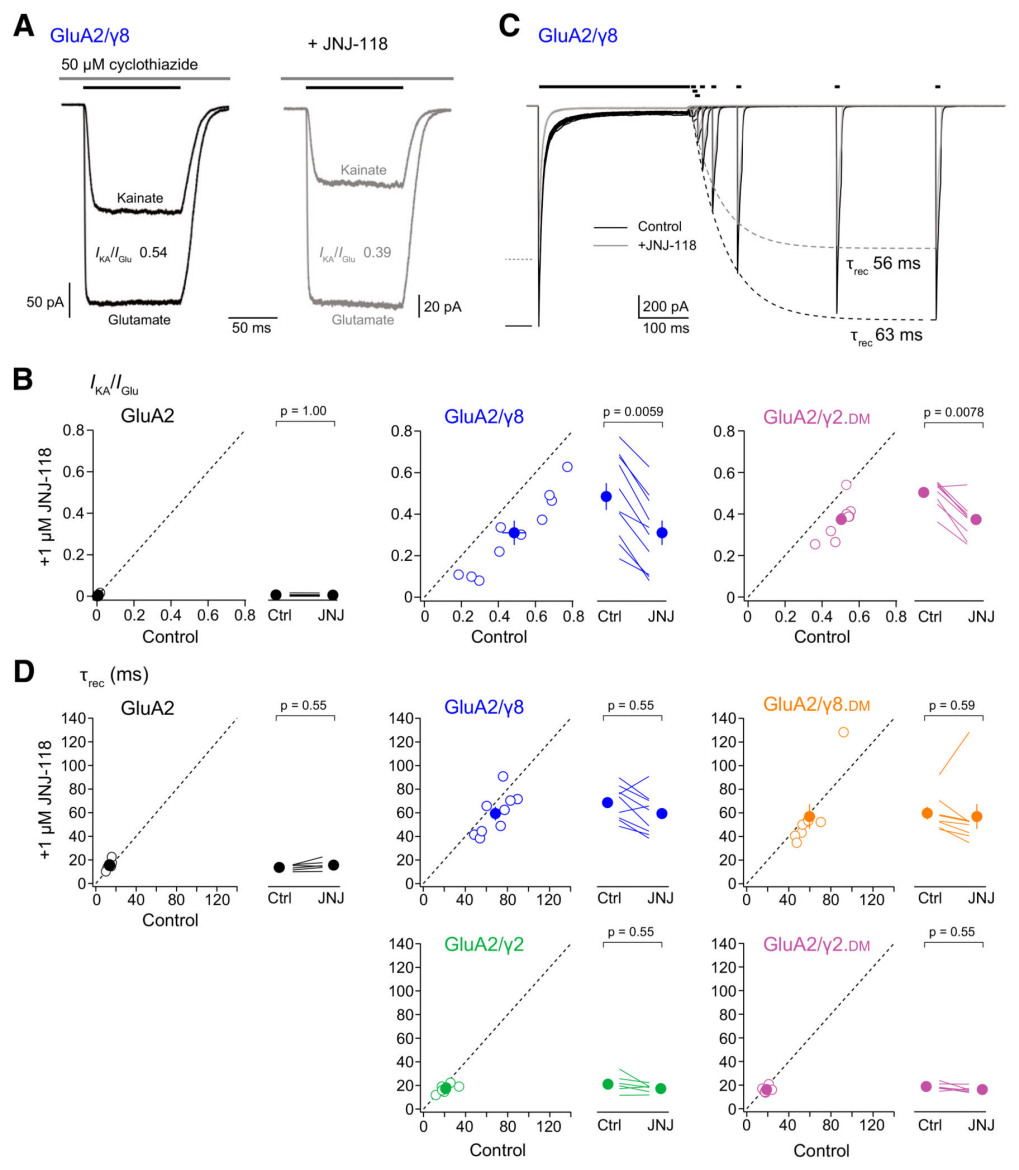
JNJ-118 influences kainate relative efficacy but not recovery from desensitization. (A) Glutamate- and kainate-evoked currents (−60 mV) recorded from the same representative patch in the presence of 50 μM cyclothiazide in the absence (left) and presence (right) of 1 μM JNJ-118. The glutamate responses are scaled to highlight the small decrease in the relative efficacy of kainate. (B) Scatter and paired plots (as in Fig. 2) showing the effects of 1 μM JNJ-118 on *I*_KA_/*I*_Glu_ for GluA2, GluA2/*γ*8, and GluA2/*γ*2.DM. Indicated *P* values are from two-sided Wilcoxon signed-rank exact tests (adjusted for multiple comparisons as described in Table 1) following a nonparametric omnibus test (Supplemental Table 1). (C) Glutamate-evoked currents (−60 mV) from a representative GluA2/*γ*8 outside-out patch demonstrating the time course of recovery following desensitization with 10 mM glutamate (250 milliseconds; black bar) in the absence and presence of 1 μM JNJ-118. Recovery of peak currents was assessed using glutamate reapplication (10 milliseconds; short black bars) at intervals from 2–500 milliseconds, and single exponentials (dashed lines) were fitted to the peak currents. (D) Scatter and paired plots showing the effects of 1 μM JNJ-118 on *τ*_rec_ for GluA2, GluA2/*γ*8, GluA2/*γ*8. DM, GluA2/*γ*2 and GluA2/*γ*2.DM. Open circles show individual values, and filled circles denote the means, with error bars indicating S.E.M. In scatter plots, dashed lines denote equality, with points below the lines indicating inhibitory effects of JNJ-118. Indicated *P* values are from two-sided Wilcoxon signed-rank exact tests (adjusted as described in Table 1) following a nonparametric omnibus test (Supplemental Table 1).

The well-documented increase in AMPAR agonist efficacy induced by TARPs is most readily seen in their effects on the action of the partial agonist kainate, specifically the kainate/glutamate current amplitude ratio (*I*_KA_/*I*_Glu_) (Tomita et al., 2005; Cho et al., 2007). We measured *I*_KA_/*I*_Glu_ for GluA2, GluA2/*γ*8, and GluA2/*γ*2.DM in the presence of cyclothiazide (Fig. 7A). For GluA2 expressed in the absence of TARP, the relative kainate efficacy was low and, as expected, JNJ-118 had no effect on *I*_KA_/*I*_Glu_ (Table 3). However, *I*_KA_/*I*_Glu_ was increased by *γ*8 and *γ*2.DM, and in both cases, it was reduced by JNJ-118 (Fig. 7B; Table 3). For both GluA2/*γ*8 and GluA2/*γ*2.DM, the relative kainate efficacy in JNJ-118 remained higher than the value seen for GluA2 alone (unpaired mean difference GluA2/*γ*8/JNJ-118 minus GluA2/JNJ-118 0.305 [0.202; 0.421] *P =* 0.0038 and GluA2/*γ*2.DM/JNJ-118 minus GluA2/JNJ-118 0.368 [0.324; 0.420] *P* = 0.0038). This finding differs from earlier work, where kainate efficacy was unaffected by JNJ-118 (Maher et al., 2016). One possible explanation for the apparent difference may be the use of cyclothiazide in our experiments, which will have minimized any influence of desensitization, thus producing a measure that solely reflected relative agonist efficacy.

As the effects of JNJ-118 on the AMPAR properties examined so far appeared consistent with a partial reversal of the modulating influence of TARPs, we also examined the effect of JNJ-118 on the recovery from desensitization of GluA2/*γ*8 (Fig. 7C) and GluA2/*γ*2.DM receptors. The effects of TARPs on the recovery of AMPARs from desensitization depend on the GluA subunit and TARP isoform. In the case of homomeric GluA2 receptors, we have shown previously that *γ*2 coexpression has little effect, whereas *γ*8 markedly slows recovery (Cais et al., 2014). Therefore, as expected, although coexpression of *γ*8 or *γ*8.DM slowed recovery from desensitization (by 4 to 5-fold), neither *γ*2 nor *γ*2.DM altered recovery kinetics of GluA2 (Table 1; Fig. 7D). Interestingly, JNJ-118 (1 μM) did not affect the recovery kinetics of either GluA2/*γ*8 or GluA2/*γ*2.DM (Table 2). Thus, of the various kinetic parameters we examined, only recovery from desensitization appeared insensitive to JNJ-118. This echoes the finding with homomeric GluA1, where the action of *γ*8 – which is known to speed recovery (Devi et al., 2020) – was also unaffected by JNJ-118 (Maher et al., 2016).

## Discussion

*γ*8-specific AMPAR inhibitors offer an exciting new avenue for the targeted treatment of various neurological and neuropsychiatric disorders as they lack side effects associated with broad-spectrum AMPAR antagonists (Gardinier et al., 2016; Kato et al., 2016; Maher et al., 2016; Maher et al., 2017). For heteromeric GluA1/2 *γ*8-containing receptors, the binding site of one of these molecules, JNJ-118, has been shown to reside between the M1 region of GluA1 and the TM3 and TM4 regions of *γ*8, suggesting the drug could act by lessening the influence of *γ*8 on AMPAR function (Maher et al., 2016; Lee et al., 2017; Dohrke et al., 2020; Yu et al., 2021). Our experiments aimed to build on this information by examining how binding of JNJ-118 changes AMPAR gating and channel behavior.

### Partial Activation of AMPAR Channels in the Presence of JNJ-118

Although the AMPAR/TARP stoichiometry of both recombinantly expressed and native receptors is variable (Shi et al., 2009; Kim et al., 2010; Twomey et al., 2016; Dawe et al., 2019), *γ*8-containing AMPARs are thought to contain two copies of the TARP, as evidenced from antibody shift assays (Schwenk et al., 2012) and cryo-EM visualization of hippocampal AMPARs (Yu et al., 2021). In native and recombinant heteromeric GluA2- and *γ*8-containing AMPARs, the GluA2 subunit occupies the gating-dominant “pore-distal” (B/D) positions, and the extracellular loops of *γ*8 (in the B’/D’ positions associated with GluA2) are thought to directly interact with the GluA2 ligand-binding domain (LBD) to modulate receptor gating (Herguedas et al., 2019; Yu et al., 2021). In the native GluA1/GluA2/*γ*8/CNIH2 receptors visualized by Yu et al. (2021), *γ*8 forms extensive contacts with both the GluA1 and GluA2 subunits in the TM regions. However, the two JNJ-118 binding sites are formed exclusively from *γ*8 and the M1 domain of GluA1. Yu et al. (2021) proposed that binding of the drug may decrease receptor activity by precluding movement of the GluA1 M1 domains away from the central axis of the channel during gating, which would thus limit dilation of the pore. Meanwhile, as the GluA2 subunits are not in direct contact with the JNJ-118 molecule, they are freer to move. This may help explain why the drug reduces, rather than eliminates, the AMPAR response.

Our results add crucial detail to the understanding of JNJ-118’s action. NSFA revealed that JNJ-118 reduces the weighted mean single-channel conductance of *γ*8-containing receptors. Further, from direct resolution of single-channel events, it was clear that the changes identified by NSFA reflected a decrease in the proportion of openings to the higher conductance sublevels. Given that the three conductance levels we identified match, respectively, the maximum conductance state O4 and sublevels O3 and O2 – produced when four, three, or two LBDs contribute to gating (Coombs et al., 2017; Coombs and Cull-Candy, 2021) – our findings suggest that JNJ-118 simply reduces the probability that any individual AMPAR subunit will “gate.” Interestingly, although maximum conductance single-channel openings were less prevalent in the presence of the drug they were clearly still detectable. This would suggest that even when JNJ-118 is bound, all subunits are capable of contributing to gating. In turn, this suggests that the drug does not prevent expansion of the “gating ring” (Yu et al., 2021) but rather reduces the extent of this expansion or the likelihood that it occurs.

The action of JNJ-118 contrasts, in certain key features, with that described for the antiepileptic noncompetitive AMPAR antagonist, perampanel. Perampanel binding sites are found on each GluA subunit (Yelshanskaya et al., 2016), and their occupancy renders the subunit unable to contribute to gating (Yuan et al., 2019). For example, when two pera-panel molecules are bound, the receptor never produces O3 or O4 openings and hence only opens to the lowest two conductance levels, whereas receptors occupied by four perampanel molecules are completely inhibited (Yuan et al., 2019). By contrast, JNJ-118 does not fully inhibit even GluA2/*γ*2.DM receptors, which likely contain four TARPs and hence four binding sites (Hastie et al., 2013). Indeed, a small proportion of openings to the highest conductance level O4 are still seen. Interestingly, however, GluA2/*γ*2.DM peak currents were inhibited to a greater extent than those of GluA2/*γ*8, possibly reflecting differences in TARP stoichiometry.

### JNJ-118 Reduces the Functional Impact of *γ*8 Incorporation

We found that although JNJ-118 application accelerated GluA2/*γ*8 deactivation and desensitization kinetics, decreased steady-state currents, decreased weighted mean conductance from NSFA, increased block by intracellular spermine, and decreased kainate efficacy, the effects were not sufficient to fully revert the properties to those of TARPless AMPARs. Our data are thus consistent with the previous suggestion that several of the changes induced in AMPARs by JNJ-118 could result from a partial disruption of the interaction between *γ*8 and GluA subunits (Maher et al., 2016).

Although the ability of JNJ-118 to reduce the proportion of single-channel openings to the higher conductance levels can be accounted for by restrictions placed on channel gating, the mechanism by which the drug accelerates deactivation kinetics and reduces the steady-state current is less apparent. TARP modulation of kinetic properties is generally viewed as an effect of the TARP’s first extracellular loop on the AMPAR LBD (Tomita et al., 2005; Turetsky et al., 2005; Cais et al., 2014; Dawe and Bowie, 2016), with additional influence from the TARP’s intracellular domains (Turetsky et al., 2005; Milstein and Nicoll, 2009). However, from cryo-EM images of hippocampal AMPARs bound to JNJ-118, it is difficult to determine how the drug might influence the LBD or the intracellular domain of the AMPAR (Yu et al., 2021). As there is tight coupling between LBD closure and channel opening (Kristensen et al., 2011; Chen et al., 2017), it follows that although deactivation/desensitization is dictated by the LBD, it will also be strongly influenced by the state of the gate. Therefore, the reduction in gating ring expansion seen in the presence of JNJ-118 (Yu et al., 2021) may well destabilize the open channel gate. This could accelerate deactivation and desensitization independent of any direct influence of JNJ-118 on the LBD.

Our observations on recovery from desensitization add further support to the idea that JNJ-118 mediates functional changes by directly influencing the channel gate rather than the LBDs. Recovery from desensitization – the transition from the desensitized to the closed state – is the only property of GluA2/*γ*8 that we found to be unaltered by JNJ-118. Recovery from desensitization involves large rearrangements of the LBD dimers which are distant from the JNJ-118 binding site, but only very subtle rearrangements of the transmembrane regions which contain the drug binding site (Chen et al., 2017; Twomey et al., 2017). Thus, unlike channel activation, deactivation, and desensitization, recovery from desensitization does not involve substantial movement (opening or closing) of the gate adjacent to the JNJ-118 binding site. Therefore, it is perhaps unsurprising that recovery from desensitization appears insensitive to JNJ-118.

### The Accessibility of the JNJ-118 Binding Site

We found no evidence that the gating state of the channel influenced JNJ-118’s ability to inhibit the currents. Thus, inhibition of open channels occurred within hundreds of milliseconds, and inhibition of closed channels was mostly complete after a single 200-millisecond application of JNJ-118, reaching equilibrium after two or three applications. This observation fits with recent structural information on the resting and active states of GluA1/GluA2/*γ*8/CNIH2 (Zhang et al., 2021), which revealed that, although gating transitions lead to expected rearrangements in the transmembrane domains, the JNJ-118 binding site is remarkably unchanged by activation.

We found that adding JNJ-118 to the intracellular solution occluded inhibition by extracellularly applied drug. This is of interest given that the JNJ-118 binding site, although found toward the extracellular side of the transmembrane regions (Yu et al., 2021; Zhang et al., 2021), appears from the cryo-EM structures to be less accessible from the extracellular space than it does from within the membrane. This raises the possibility that JNJ-118 could access its binding site through the membrane’s lipid phase even when applied from the outside.

The *γ*8-selective blockers represent an exciting development for the treatment of epilepsy. Most obviously, given their selective inhibition of forebrain AMPARs, they offer the promise of reduced motor side-effects (Zwart et al., 2014; Kato et al., 2016; Maher et al., 2016). However, the nonselective negative allosteric modulator perampanel, particularly at higher doses, additionally causes mood disturbance including depression and aggression (Ettinger et al., 2015; Villanueva et al., 2021). The action of JNJ-118 that we have identified – a reduction of single-channel conductance rather than a complete block – might suggest a further potential benefit of *γ*8-selective drugs. By producing partial inhibition of forebrain AMPARs, *γ*8-selective drugs such as JNJ-118 may enable a more nuanced intervention that could help to limit behavioral side-effects.

## Supplementary Material

Supplementary Materials

## Figures and Tables

**Table 1:** Peak current block by JNJ-118 of GluA2 coexpressed with wild-type or mutated forms of *γ*8 and *γ*2 Summary of peak current block (*I*_118_/*I*_Ctrl_), presented as mean ± S.D. from (n) patches. To assess the extent of inhibition, one-sample Wilcoxon signed-rank tests (against 100%) were used. For TARP effects, unpaired mean differences (upMD; GluA2+TARP versus GluA2 alone) with 95% confidence intervals (lower bound, upper bound), and *P* values from Wilcoxon rank-sum tests are shown. For pairwise tests, the *P* values were adjusted for multiple comparisons using Holm’s sequential Bonferroni correction.

	*I*_118_*/I*_Ctrl_ (%)	Compared with 100%	Compared with GluA2	
		*P* value	upMD [95% CI]	P value
GluA2	94.5 ± 19.5 (16)	0.77	–	–
GluA2/γ8	59.5 ± 12.9 (19)	<0.0001	−35.0 [−46.8, −25.1]	<0.0001
GluA2/γ8.DM	96.6 ± 12.9 (16)	0.77	2.1 [−10.1, 12.2]	0.54
GluA2/γ2	98.2 ± 7.2 (18)	0.77	3.7 [−6.5, 13.0]	0.18
GluA2/γ2.DM	45.8 ± 8.7 (19)	<0.0001	−48.8 [−59.5, −39.7]	<0.0001

**Table 2:** Actions of JNJ-118 on kinetics of GluA2 coexpressed with wild-type or mutated forms of *γ*8 and *γ*2 Summary data for measures of current deactivation and desensitization (*τ*_w, deact_, *τ*_w, des_, *I*_ss_/*I*_peak_, and *τ*_rec_) presented as mean ± S.D. from (n) patches. Also shown are unpaired or paired mean differences (upMD and pMD) with 95% confidence intervals (lower bound, upper bound) and *P* values from two-sample tests. For TARP effects, Wilcoxon rank-sum tests were used for unpaired comparisons (GluA2+TARP versus GluA2 alone). For the drug effects, Wilcoxon signed-rank tests were used for paired comparisons (JNJ-118 versus corresponding control). For pairwise tests, the *P* values were adjusted for multiple comparisons within each family of comparisons (for each different measure) using Holm’s sequential Bonferroni correction.

		TARP Effect			Drug Effect	
	Control	upMD [95% CI]	*P* value	+JNJ-118	pMD [95% CI]	P value
τ_w,deact_ (ms)						
GluA2	0.41 ± 0.07 (7)	–	–	0.45 ± 0.13 (7)	0.044 [−0.003, 0.17]	0.48
GluA2/γ8	1.34 ± 0.61 (9)	0.93 [0.64, 1.45]	0.0019	0.93 ± 0.47 (9)	−0.42 [−0.58, −0.27]	0.023
GluA2/γ8.DM	1.12 ± 0.58 (7)	0.72 [0.45, 1.36]	0.0052	1.05 ± 0.52 (7)	−0.071 [−0.14, 0.007]	0.48
GluA2/γ2	1.98 ± 1.87 (10)	1.57 [0.67, 2.88]	0.0012	1.79 ± 1.50 (10)	−0.19 [−0.5, 0.018]	0.48
GluA2/γ2.DM	1.34 ± 1.35 (12)	0.93 [0.47, 2.35]	0.010	0.3 ± 0.98 (12)	−0.41 [−0.74, −0.26]	0.0049
						
τ_w,des_ (ms)						
GluA2	5.6 ± 0.9 (16)	–	–	5.0 ± 1.0 (16)	−0.54 [−1.19, 0.12]	0.30
GluA2/γ8	10.6 ± 3.1 (19)	5.04 [3.81, 6.75]	<0.0001	7.8 ± 2.1 (19)	−2.78 [−4.74, −1.39]	<0.0001
GluA2/γ8.DM	10.3 ± 5.1 (16)	4.78 [2.88, 8.17]	<0.0001	9.7 ± 3.5 (16)	−0.62[−4.10, 1.88]	1.00
GluA2/γ2	12.3 ± 5.0 (18)	6.75 [4.81, 9.4]	<0.0001	12.3 ± 6.0 (18)	0.017[−3.07, 4.13]	1.00
GluA2/γ2.DM	12.0 ± 5.0 (19)	6.4 [4.63, 9.47]	<0.0001	5.5 ± 1.7 (19)	−6.44 [−9.61, −4.60]	<0.0001
						
*I*_ss_/*I*_peak_ (%)						
GluA2	1.17 ± 1.16 (16)	–	–	0.90 ± 0.65 (16)	−0.27 [−0.68, −0.023]	0.65
GluA2/γ8	4.51 ± 4.02 (19)	3.35 [1.73, 5.53]	0.0047	2.74 ± 1.83 (19)	−1.78 [−3.32, −0.78]	0.049
GluA2/γ8.DM	4.88 ± 6.23 (16)	3.71 [1.55, 8.56]	0.022	4.30 ± 5.74 (16)	−0.58 [−1.49, 0.074]	0.53
GluA2/γ2	8.09 ± 6.55 (18)	6.92 [4.78, 11.6]	<0.0001	7.95 ± 5.89 (18)	−0.14 [−1.19, 0.86]	0.83
GluA2/γ2.DM	6.98 ± 4.42 (19)	5.81 [4.05, 8.11]	<0.0001	3.26 ± 2.31 (19)	−3.72 [−5.47, −2.05]	0.0047
						
τ_rec _(ms)						
GluA2	13.6 ± 2.5 (6)	–	–	15.6 ± 4.1 (6)	2.02 [0.61, 4.37]	0.55
GluA2/γ8	69.8 ± 14.1 (10)	56.2 [47.3, 64.3]	0.0032	62.1 ± 18.5 (10)	−7.8 [−14.3, 0.62]	0.55
GluA2/γ8.DM	59.7 ± 15.2 (8)	46.1 [38.8, 60.4]	0.0073	56.9 ± 29.6 (8)	−2.85 [−9.9, 15.3]	0.59
GluA2/γ2	21.1 ± 7.1 (7)	7.47 [2.67, 12.8]	0.13	17.3 ± 3.4 (7)	−3.77 [−9.09, −1.16]	0.55
GluA2/γ2.DM	18.8 ± 3.2 (6)	5.22 [2.47, 8.38]	0.13	16.2 ± 2.5 (6)	−2.59 [−5.16, −0.25]	0.55

**Table 3:** Actions of JNJ-118 on rectification and conductance of GluA2 coexpressed with wild-type or mutated forms of *γ*8 and *γ*2 Summary data for measures of current rectification (*V*_b_ and RI), channel properties (*γ* and *P*_o, peak_), and kainate efficacy (*I*_KA_/*I*_Glu_) presented as mean ± S.D. from (n) patches. Also shown are unpaired or paired mean differences (upMD and pMD) with 95% confidence intervals (lower bound, upper bound) and *P* values from two-sample tests. Details as for Table 2.

			TARP Effect			Drug Effect	
	Control	upMD [95% CI]		*P* value	+JNJ-118	pMD [95% CI]	*P* value
*V*_b_** **(mV)							
GluA2	−60.8 ± 4.7 (6)	–		–	−65.2 ± 8.0 (6)	−4.37 [−8.65, −0.38]	0.47
GluA2/γ8	−33.1 ± 5.5 (8)	27.7 [22.7, 32.7]		0.0087	−41.1 ± 4.4 (8)	−8.05 [−11.0, −6.34]	0.039
GluA2/γ8.DM	−45.5 ± 3.4 (7)	15.4 [11.0, 19.4]		0.010	−46.6 ± 3.6 (7)	−1.11 [−4.51, 1.96]	0.69
GluA2/γ2	−25.3 ± 9.7 (6)	35.5 [24.7, 41.5]		0.015	−28.1 ± 8.3 (6)	−2.79 [−5.38, 0.34]	0.47
GluA2/γ2.DM	−28.1 ± 5.9 (8)	32.8 [27.3, 37.7]		0.0087	−41.1 ± 6.8 (8)	−13.0 [−15.3, −11.4]	0.039
RI (*I*_+60_/*I*_−60_)							
GluA2	0.04 ± 0.03 (5)	–		–	0.06 ± 0.04 (5)	0.013 [−0.020, 0.070]	1.00
GluA2/γ8	0.37 ± 0.21 (8)	0.33 [0.22, 0.51]		0.020	0.14 ± 0.08 (8)	−0.24 [−0.35, −0.16]	0.055
GluA2/γ8.DM	0.25 ± 0.10 (7)	0.20 [0.14, 0.28]		0.028	0.24 ± 0.08 (7)	−0.007 [−0.065, 0.053]	1.00
GluA2/γ2	0.44 ± 0.21 (6)	0.40 [0.27, 0.60]		0.039	0.45 ± 0.25 (6)	0.013 [−0.025, 0.071]	1.00
GluA2/γ2.DM	0.32 ± 0.09 (8)	0.27 [0.22, 0.34]		0.020	0.10 ± 0.05 (8)	−0.21 [−0.27, −0.15]	0.055
γ (pS)							
GluA2	17.3 ± 4.4 (9)	–		–	17.9 ± 4.2 (9)	0.64 [−1.71, 2.71]	1.00
GluA2/γ8	32.1 ± 7.6 (9)	14.7 [9.18, 20.0]		0.0035	20.6 ± 4.7 (9)	−11.4 [−14.7, −8.3]	0.028
GluA2/γ8.DM	27.9 ± 7.3 (8)	10.6 [5.77, 17.0]		0.0097	29.0 ± 7.4 (8)	1.1 [−3.3, 6.9]	1.00
GluA2/γ2	28.5 ± 8.3 (10)	11.2 [6.2, 17.9]		0.0097	28.4 ± 4.3 (10)	−0.051 [−2.95, 3.67]	1.00
GluA2/γ2.DM	26.2 ± 9.7 (13)	8.88 [3.32, 14.7]		0.10	18.1 ± 6.6 (13)	−8.10 [−11.50, −5.49]	0.0054
*P*_o, peak_							
GluA2	0.33 ± 0.22 (9)	–		–	0.34 ± 0.11 (9)	0.0050 [−0.092, 0.080]	1.00
GluA2/γ8	0.68 ± 0.12 (9)	0.35 [0.19, 0.49]		0.011	0.52 ± 0.15 (9)	−0.16 [−0.27, −0.043]	0.14
GluA2/γ8.DM	0.59 ± 0.17 (8)	0.25 [0.061, 0.41]		0.19	0.57 ± 0.19 (8)	−0.020 [−0.14, 0.15]	1.00
GluA2/γ2	0.77 ± 0.15 (10)	0.44 [0.25, 0.58]		0.0045	0.72 ± 0.16 (10)	−0.05 [−0.090, 0.0017]	0.32
GluA2/γ2.DM	0.76 ± 0.07 (13)	0.43 [0.27, 0.55]		<0.0001	0.52 ± 0.14 (13)	−0.24 [−0.28, −0.18]	0.0029
*I*_KA_ */I*_Glu_							
GluA2	0.006 ± 0.005 (7)	–		–	0.005 ± 0.006 (7)	−0.001 [−0.004,0.0002]	1.00
GluA2/γ8	0.49 ± 0.20 (10)	0.48 [0.36, 0.60]		0.00072	0.31 ± 0.19 (10)	−0.18 [−0.21, −0.13]	0.0059
GluA2/γ2.DM	0.50 ± 0.06 (10)	0.50 [0.45, 0.53]		0.00072	0.37 ± 0.08 (10)	−0.13 [−0.15, −0.082]	0.0078

## Significance Statement

Although modulation of AMPA-type glutamate receptors shows promise for the treatment various neurological conditions, the absence of subtype-selective drugs has hindered adoption of this therapeutic strategy. We made patch-clamp recordings to characterize the actions of the γ8-selective AMPAR inhibitor JNJ-55511118 on GluA2(Q) receptors expressed in HEK cells. We report that JNJ-55511118 inhibits AMPAR-mediated currents by reducing single-channel conductance, providing clear insight into the mechanism of action of this important class of AMPAR modulators.

## Abbreviations

AMPA: *α*-amino-3-hydroxy-5-methyl-4-isoxazolepropionic acid
AMPAR: AMPA receptor
EM: electron microscopy
*I*_peak_: peak current
*I*_ss_: steady-state current
JNJ-118: JNJ-55511118
LBD: ligand-binding domain
NSFA: nonstationary fluctuation analysis
*P*_o,peak_: peak open probability
pS: picosiemens
RI: rectification index
TARP: transmembrane AMPAR regulatory protein
TM: transmembrane.

## References

[R1] Bowie D, Lange GD, Mayer ML (1998). Activity-dependent modulation of glutamate receptors by polyamines. J Neurosci.

[R2] Brogi S, Campiani G, Brindisi M, Butini S (2019). Allosteric modulation of ionotropic glutamate receptors: an outlook on new therapeutic approaches to treat central nervous system disorders. ACS Med Chem Lett.

[R3] Brown PMGE, McGuire H, Bowie D (2018). Stargazin and cornichon-3 relieve polyamine block of AMPA receptors by enhancing blocker permeation. J Gen Physiol.

[R4] Cais O, Herguedas B, Krol K, Cull-Candy SG, Farrant M, Greger IH (2014). Mapping the interaction sites between AMPA receptors and TARPs reveals a role for the receptor N-terminal domain in channel gating. Cell Rep.

[R5] Carbone AL, Plested AJ (2016). Superactivation of AMPA receptors by auxiliary proteins. Nat Commun.

[R6] Carrillo ES, Shaikh A, Berka V, Durham RJ, Litwin DB, Lee G, MacLean DM, Nowak LM, Jayaraman V (2019). Mechanism of modulation of AMPA receptors by TARP-γ8. J Gen Physiol.

[R7] Chen S, Zhao Y, Wang Y, Shekhar M, Tajkhorshid E, Gouaux E (2017). Activation and desensitization mechanism of AMPA receptor-TARP complex by cryo-EM. Cell.

[R8] Cho CH, St-Gelais F, Zhang W, Tomita S, Howe JR (2007). Two families of TARP isoforms that have distinct effects on the kinetic properties of AMPA receptors and synaptic currents. Neuron.

[R9] Coombs ID, MacLean DM, Jayaraman V, Farrant M, Cull-Candy SG (2017). Dual Effects of TARP γ-2 on Glutamate Efficacy Can Account for AMPA Receptor Autoinactivation. Cell Rep.

[R10] Coombs ID, Soto D, Zonouzi M, Renzi M, Shelley C, Farrant M, Cull-Candy SG (2012). Cornichons modify channel properties of recombinant and glial AMPA receptors. J Neurosci.

[R11] Coombs ID, Bats C, Sexton CA, Cull-Candy SG, Farrant M (2021). Intracellular NASPM allows an unambiguous functional measure of GluA2-lacking calcium-permeable AMPA receptor prevalence. bioRxiv.

[R12] Coombs ID, Cull-Candy SG (2021). Single-channel mechanisms underlying the function, diversity and plasticity of AMPA receptors. Neuropharmacology.

[R13] Dawe GB, Bowie D (2016). Mechanism of AMPA receptor gating re-shaped by auxiliary proteins. Biophys J.

[R14] Dawe GB, Kadir MF, Venskutonytė R, Perozzo AM, Yan Y, Alexander RPD, Navarrete C, Santander EA, Arsenault M, Fuentes C (2019). Nanoscale mobility of the apo state and TARP stoichiometry dictate the gating behavior of alternatively spliced AMPA receptors. Neuron.

[R15] Devi SPS, Cheng Y, Tomita S, Howe JR, Zhang W (2020). TARPs modulate receptor-mediated paired-pulse depression and recovery from desensitization. J Neurosci.

[R16] Dohrke JN, Watson JF, Birchall K, Greger IH (2020). Characterizing the binding and function of TARP γ8-selective AMPA receptor modulators. J Biol Chem.

[R17] Ettinger AB, LoPresti A, Yang H, Williams B, Zhou S, Fain R, Laurenza A (2015). Psychiatric and behavioral adverse events in randomized clinical studies of the non-competitive AMPA receptor antagonist perampanel. Epilepsia.

[R18] Frydenvang K, Pickering DS, Kastrup JS (2022). Structural basis for positive allosteric modulation of AMPA and kainate receptors. J Physiol.

[R19] Fukaya M, Yamazaki M, Sakimura K, Watanabe M (2005). Spatial diversity in gene expression for VDCCy subunit family in developing and adult mouse brains. Neurosci Res.

[R20] Gardinier KM, Gernert DL, Porter WJ, Reel JK, Ornstein PL, Spinazze P, Stevens FC, Hahn P, Hollinshead SP, Mayhugh D (2016). Discovery of the first α-amino-3-hydroxy-5-methyl-4-isoxazolepropionic acid (AMPA) receptor antagonist dependent upon transmembrane AMPA receptor regulatory protein (TARP) γ-8. J Med Chem.

[R21] Hanada T (2020). Ionotropic glutamate receptors in epilepsy: a review focusing on AMPA and NMDA receptors. Biomolecules.

[R22] Hansen KB, Wollmuth LP, Bowie D, Furukawa H, Menniti FS, Sobolevsky AI, Swanson GT, Swanger SA, Greger IH, Nakagawa T (2021). Structure, function, and pharmacology of glutamate receptor ion channels. Pharmacol Rev.

[R23] Hastie P, Ulbrich MH, Wang HL, Arant RJ, Lau AG, Zhang Z, Isacoff EY, Chen L (2013). AMPA receptor/TARP stoichiometry visualized by single-molecule subunit counting. Proc Natl Acad Sci USA.

[R24] Herguedas B, Watson JF, Ho H, Cais O, Garc’ıa-Nafr’ıa J, Greger IH (2019). Architecture of the heteromeric GluA1/2 AMPA receptor in complex with the auxiliary subunit TARP γ8. Science.

[R25] Ho J, Tumkaya T, Aryal S, Choi H, Claridge-Chang A (2019). Moving beyond P values: data analysis with estimation graphics. Nat Methods.

[R26] Ishii T, Stolz JR, Swanson GT (2020). Auxiliary proteins are the predominant determinants of differential efficacy of clinical candidates acting as AMPA receptor positive allosteric modulators. Mol Pharmacol.

[R27] Jackson AC, Nicoll RA (2011). The expanding social network of ionotropic glutamate receptors: TARPs and other transmembrane auxiliary subunits. Neuron.

[R28] Kato AS, Burris KD, Gardinier KM, Gernert DL, Porter WJ, Reel J, Ding C, Tu Y, Schober DA, Lee MR (2016). Forebrain-selective AMPA-receptor antagonism guided by TARP γ-8 as an antiepileptic mechanism. Nat Med.

[R29] Kato AS, Gill MB, Ho MT, Yu H, Tu Y, Siuda ER, Wang H, Qian Y-W, Nisenbaum ES, Tomita S (2010). Hippocampal AMPA receptor gating controlled by both TARP and cornichon proteins. Neuron.

[R30] Kim KS, Yan D, Tomita S (2010). Assembly and stoichiometry of the AMPA receptor and transmembrane AMPA receptor regulatory protein complex. J Neurosci.

[R31] Kristensen AS, Jenkins MA, Banke TG, Schousboe A, Makino Y, Johnson RC, Huganir R, Traynelis SF (2011). Mechanism of Ca^2+^/calmodulin-dependent kinase II regulation of AMPA receptor gating. Nat Neurosci.

[R32] Lee MR, Gardinier KM, Gernert DL, Schober DA, Wright RA, Wang H, Qian Y, Witkin JM, Nisenbaum ES, Kato AS (2017). Structural determinants of the γ-8 TARP dependent AMPA receptor antagonist. ACS Chem Neurosci.

[R33] Lynch G (2006). Glutamate-based therapeutic approaches: ampakines. Curr Opin Pharmacol.

[R34] Maher MP, Wu N, Ravula S, Ameriks MK, Savall BM, Liu C, Lord B, Wyatt RM, Matta JA, Dugovic C (2016). Discovery and characterization of AMPA receptor modulators selective for TARP-γ8. J Pharmacol Exp Ther.

[R35] Maher MP, Matta JA, Gu S, Seierstad M, Bredt DS (2017). Getting a handle on neuropharmacology by targeting receptor-associated proteins. Neuron.

[R36] Matthews PM, Pinggera A, Kampjut D, Greger IH (2021). Biology of AMPA receptor interacting proteins - from biogenesis to synaptic plasticity. Neuropharmacology.

[R37] Milstein AD, Nicoll RA (2009). TARP modulation of synaptic AMPA receptor trafficking and gating depends on multiple intracellular domains. Proc Natl Acad Sci USA.

[R38] Noguchi K, Gel YR, Brunner E, Konietschke F (2012). nparLD: an R software package for the nonparametric analysis of longitudinal data in factorial experiments. J Stat Softw.

[R39] Panchenko VA, Glasser CR, Partin KM, Mayer ML (1999). Amino acid substitutions in the pore of rat glutamate receptors at sites influencing block by polyamines. J Physiol.

[R40] Partin KM (2015). AMPA receptor potentiators: from drug design to cognitive enhancement. Curr Opin Pharmacol.

[R41] Pollard KS, Dudoit S, van der Laan MJ, Gentleman R, Carey V, Huber W, Irizarry R, Dudoit S (2005). Bioinformatics and Computational Biology Solutions Using R and Bioconductor.

[R42] Potschka H, Trinka E (2019). Perampanel: Does it have broad-spectrum potential?. Epilepsia.

[R43] Ravula S, Savall BM, Wu N, Lord B, Coe K, Wang K, Seierstad M, Swanson DM, Ziff J, Nguyen M (2018). Lead optimization of 5-aryl benzimidazolone-and oxindole-based AMPA receptor modulators selective for TARP γ-8. ACS Med Chem Lett.

[R44] Riva I, Eibl C, Volkmer R, Carbone AL, Plested AJ (2017). Control of AMPA receptor activity by the extracellular loops of auxiliary proteins. eLife.

[R45] Rogawski MA (2011). Revisiting AMPA receptors as an antiepileptic drug target. Epilepsy Curr.

[R46] Rouach N, Byrd K, Petralia RS, Elias GM, Adesnik H, Tomita S, Karimzadegan S, Kealey C, Bredt DS, Nicoll RA (2005). TARP γ-8 controls hippocampal AMPA receptor number, distribution and synaptic plasticity. Nat Neurosci.

[R47] Savall BM, Wu D, Swanson DM, Seierstad M, Wu N, Vives Martinez J, García Olmos B, Lord B, Coe K, Koudriakova T (2019). Discovery of imidazo[1,2-a]pyrazines and pyrazolo[1,5-c]pyrimidines as TARP γ-8 selective AMPAR negative modulators. ACS Med Chem Lett.

[R48] Schwenk J, Harmel N, Brechet A, Zolles G, Berkefeld H, Müller CS, Bildl W, Baehrens D, Hüber B, Kulik A (2012). High-resolution proteomics unravel architecture and molecular diversity of native AMPA receptor complexes. Neuron.

[R49] Shelley C, Farrant M, Cull-Candy SG (2012). TARP-associated AMPA receptors display an increased maximum channel conductance and multiple kinetically distinct open states. J Physiol.

[R50] Shi Y, Lu W, Milstein AD, Nicoll RA (2009). The stoichiometry of AMPA receptors and TARPs varies by neuronal cell type. Neuron.

[R51] Soto D, Coombs ID, Kelly L, Farrant M, Cull-Candy SG (2007). Stargazin attenuates intracellular polyamine block of calcium-permeable AMPA receptors. Nat Neurosci.

[R52] Soto D, Coombs ID, Renzi M, Zonouzi M, Farrant M, Cull-Candy SG (2009). Selective regulation of long-form calcium-permeable AMPA receptors by an atypical TARP, γ-5. Nat Neurosci.

[R53] Stenum-Berg C, Musgaard M, Chavez-Abiega S, Thisted CL, Barrella L, Biggin PC, Kristensen AS (2019). Mutational analysis and modeling of negative allosteric modulator binding sites in AMPA receptors. Mol Pharmacol.

[R54] Tomita S, Adesnik H, Sekiguchi M, Zhang W, Wada K, Howe JR, Nicoll RA, Bredt DS (2005). Stargazin modulates AMPA receptor gating and trafficking by distinct domains. Nature.

[R55] Tsai J-J, Wu T, Leung H, Desudchit T, Tiamkao S, Lim K-S, Dash A (2018). Perampanel, an AMPA receptor antagonist: From clinical research to practice in clinical settings. Acta Neurol Scand.

[R56] Turetsky D, Garringer E, Patneau DK (2005). Stargazin modulates native AMPA receptor functional properties by two distinct mechanisms. J Neurosci.

[R57] Twomey EC, Yelshanskaya MV, Grassucci RA, Frank J, Sobolevsky AI (2016). Elucidation of AMPA receptor-stargazin complexes by cryo-electron microscopy. Science.

[R58] Twomey EC, Yelshanskaya MV, Grassucci RA, Frank J, Sobolevsky AI (2017). Structural bases of desensitization in AMPA receptor-auxiliary subunit complexes. Neuron.

[R59] Villanueva V, D’Souza W, Goji H, Kim DW, Liguori C, McMurray R, Najm I, Santamarina E, Steinhoff BJ, Vlasov P, PERMIT pooled analysis participants (2021). PERMIT study: a global pooled analysis study of the effectiveness and tolerability of perampanel in routine clinical practice. J Neurol.

[R60] Yelshanskaya MV, Singh AK, Sampson JM, Narangoda C, Kurnikova M, Sobolevsky AI (2016). Structural bases of noncompetitive inhibition of AMPA-subtype ionotropic glutamate receptors by antiepileptic drugs. Neuron.

[R61] Yu J, Rao P, Clark S, Mitra J, Ha T, Gouaux E (2021). Hippocampal AMPA receptor assemblies and mechanism of allosteric inhibition. Nature.

[R62] Yuan CL, Shi EY, Srinivasan J, Ptak CP, Oswald RE, Nowak LM (2019). Modulation of AMPA receptor gating by the anticonvulsant drug, perampanel. ACS Med Chem Lett.

[R63] Zhang D, Watson JF, Matthews PM, Cais O, Greger IH (2021). Gating and modulation of a hetero-octameric AMPA glutamate receptor. Nature.

[R64] Zwart R, Sher E, Ping X, Jin X, Sims JR, Chappell AS, Gleason SD, Hahn PJ, Gardinier K, Gernert DL (2014). Perampanel, an antagonist of α-amino-3-hydroxy-5-methyl-4-isoxazolepropionic acid receptors, for the treatment of epilepsy: studies in human epileptic brain and nonepileptic brain and in rodent models. J Pharmacol Exp Ther.

