# Supplementary material for: Influence of the TARP γ8-Selective Negative Allosteric Modulator JNJ-55511118 on AMPA Receptor Gating and Channel Conductance
Source: Mol Pharmacol. Author manuscript; Available in PMC 2024 Apr 2. (PMC7615793; doi:10.1124/molpharm.121.000473)
Supplement: Supplementary Materials [file EMS194668-supplement-Supplementary_Materials.pdf]

Influence of the TARP γ8-selective negative allosteric modulator JNJ-55511118 on AMPA receptor gating and channel conductance

Ian D. Coombs, Craig A. Sexton, Stuart G. Cull-Candy, Mark Farrant

Molecular Pharmacology   MOLPHARM-AR-2021-000473

Fig S1. JNJ-118 effects on kinetics and steady-state current of GluA2(Q)/γ8.

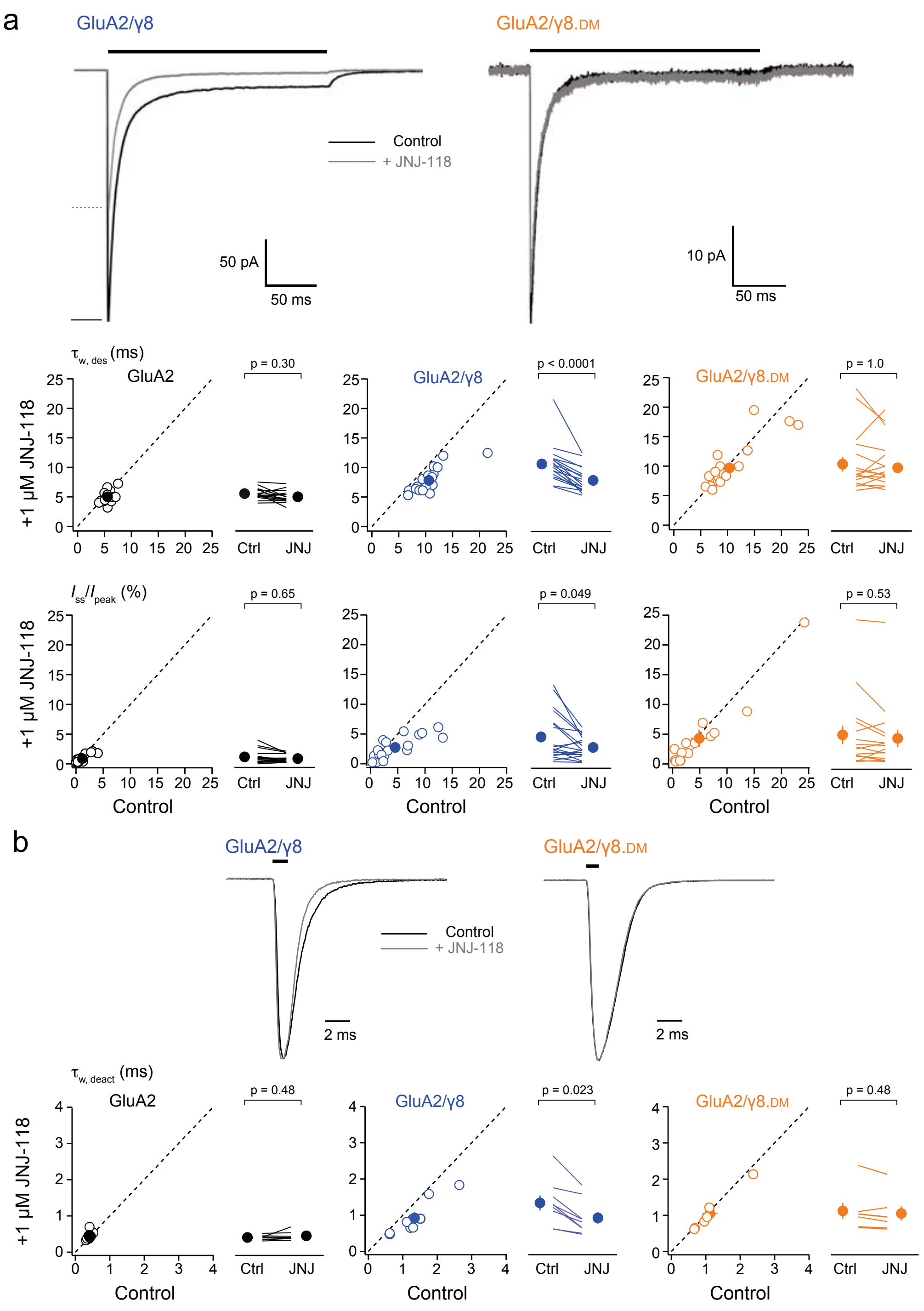

Figure S1. JNJ-118 effects on kinetics and steady-state current of GluA2(Q)/γ8

a) Representative outside-out patch responses (10 mM glutamate, 200 ms) (black bars) recorded at −60 mV from HEK293 cells transfected with GluA2/γ8 (left) or GluA2/γ8.DM (right) in control conditions (black) or in the presence of 1 µM JNJ-118 (grey). Lower panels are scatter and paired plots (as in **Fig 2**) showing the effects of JNJ-118 on the weighted mean time constant of desensitization ($\tau_{w,\ des}$) and fractional steady-state component (I_{ss}/I_{peak}) for GluA2, GluA2/γ8 and GluA2/γ8.DM. Indicated p-values (adjusted for multiple comparisons as described in **Table 1**) are from two-sided Wilcoxon signed rank exact tests following a non-parametric omnibus test (**Table S1**). **b**) Representative outside-out patch responses (10 mM

glutamate, 1 ms) (black bars) recorded at −60 mV from HEK293 cells transfected with GluA2/γ8 (left) or GluA2/γ8.DM (right) in control conditions (black) or in the presence of 1 μM JNJ-118 (grey). Lower panels are scatter and paired plots showing the effects of JNJ-118 on the weighted mean time constant of deactivation ($\tau_{w,\ deact}$) for GluA2, GluA2/γ8 and GluA2/γ8.DM. Indicated p-values (adjusted as described in **Table 1**) are from two-sided Wilcoxon signed rank exact tests following a non-parametric omnibus test (**Table S1**).

Influence of the TARP γ8-selective negative allosteric modulator JNJ-55511118 on AMPA receptor gating and channel conductance

Ian D. Coombs, Craig A. Sexton, Stuart G. Cull-Candy, Mark Farrant

Molecular Pharmacology   MOLPHARM-AR-2021-000473

Fig S2. JNJ-118 effects on deactivation and rectification of GluA2(Q)/γ2 and GluA2(Q)/γ2.DM.

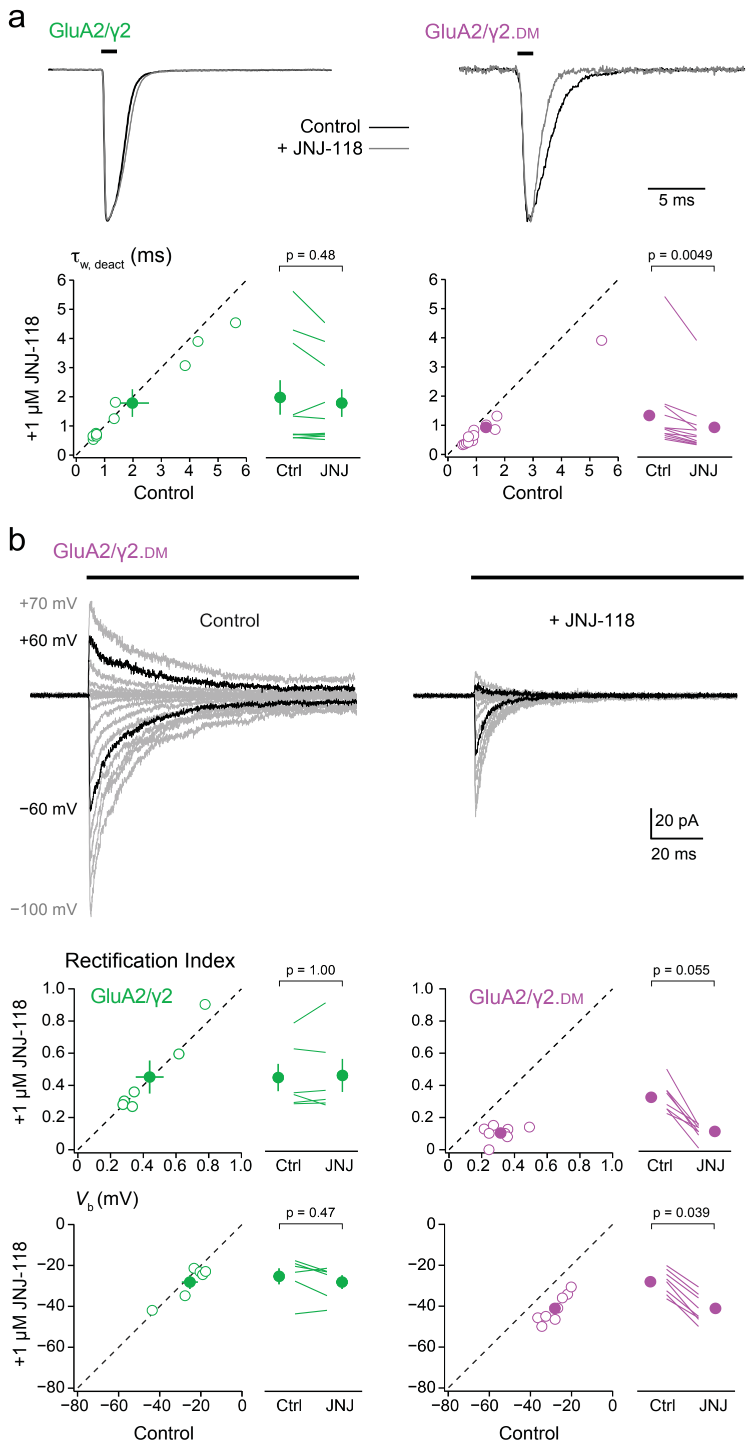

Figure S2. JNJ-118 effects on deactivation and rectification of GluA2(Q)/γ2 and GluA2(Q)/γ2.DM

a) Representative outside-out patch responses (10 mM glutamate, 1 ms) (black bars) recorded at −60 mV from HEK293 cells transfected with GluA2/γ2 (left) or GluA2/γ2.DM (right) in control conditions (black) or in the presence of 1 µM JNJ-118 (grey). Lower panels are scatter and paired plots showing the effects of JNJ-118 on the weighted mean time constant of deactivation ($\tau_{w,\ deact}$). Indicated p-values (adjusted for multiple comparisons as described in **Table 1**) are from two-sided Wilcoxon signed rank exact tests following a non-parametric

omnibus test (**Table S1**). **b**) Representative responses evoked by 10 mM glutamate (200 ms; black bars) (as in **Fig 2**) showing the effects of 1 μM JNJ-118 on Rectification Index and V_b (from individual double Boltzmann fitted conductance-voltage relationships) for GluA2/γ2 and GluA2/γ2.DM. Indicated p-values (adjusted as described in **Table 1**) are from two-sided Wilcoxon signed rank exact tests following a non-parametric omnibus test (**Table S1**).

Influence of the TARP γ8-selective negative allosteric modulator JNJ-55511118 on AMPA receptor gating and channel conductance

Ian D. Coombs, Craig A. Sexton, Stuart G. Cull-Candy, Mark Farrant

Molecular Pharmacology   MOLPHARM-AR-2021-000473

Table S1. Omnibus tests for actions of JNJ-118 on GluA2 co-expressed with wild- type or mutated forms of γ8 and γ2.

Measure	Test	Condition	Statistic	df	p-value	Figure
I_{118}/I_{Ctrl} (%)	nparLD	Main effect of TARP	33.41	2.67	<0.0001	1b and 6b
		Main effect of drug	59.98	1.00	<0.0001	
		Interaction TARP:drug	11.48	2.73	<0.0001	
$\tau_{w, deact}$ (ms)	nparLD	Main effect of TARP	8.23	3.52	<0.0001	S1b and S2a
		Main effect of drug	26.52	1.00	<0.0001	
		Interaction TARP:drug	10.24	3.10	<0.0001	
$\tau_{w, des}$ (ms)	nparLD	Main effect of TARP	24.26	3.44	<0.0001	6c and S1a
		Main effect of drug	94.82	1.00	<0.0001	
		Interaction TARP:drug	33.96	3.07	<0.0001	
I_{ss}/I_{peak} (%)	nparLD	Main effect of TARP	17.49	3.02	<0.0001	6c and S1a
		Main effect of drug	21.01	1.00	<0.0001	
		Interaction TARP:drug	6.96	3.33	<0.0001	
γ (pS)	nparLD	Main effect of TARP	8.43	3.72	<0.0001	2b and 6c
		Main effect of drug	16.59	1.00	<0.0001	
		Interaction TARP:drug	10.89	3.24	<0.0001	
P_{open}	nparLD	Main effect of TARP	11.50	3.38	<0.0001	2b and 6c
		Main effect of drug	23.67	1.00	<0.0001	
		Interaction TARP:drug	4.77	2.86	0.0029	
RI (I_{+60}/I_{-60})	nparLD	Main effect of TARP	22.11	3.28	<0.0001	4c and S2b
		Main effect of drug	40.26	1.00	<0.0001	
		Interaction TARP:drug	15.07	3.09	<0.0001	
V_b (mV)	nparLD	Main effect of TARP	33.41	2.67	<0.0001	4c and S2b
		Main effect of drug	59.98	1.00	<0.0001	
		Interaction TARP:drug	11.48	2.72	<0.0001	
JNJ-118_{int} inhib. (%)	K-W		11.96	2.00	0.0025	5d
I_{KA}/I_{Glu}	nparLD	Main effect of TARP	33.54	1.49	<0.0001	7b
		Main effect of drug	59.25	1.00	<0.0001	
		Interaction TARP:drug	11.36	1.82	<0.0001	
$\tau_{w, rec}$ (ms)	nparLD	Main effect of TARP	36.89	3.80	<0.0001	7d
		Main effect of drug	8.94	1.00	0.028	
		Interaction TARP:drug	3.31	3.21	0.017	

Table S1. Omnibus tests for actions of JNJ-118 on GluA2 co-expressed with wild-type or mutated forms of γ8 and γ2.

Omnibus tests performed prior to the pairwise statistical analyses presented in **Table 1** and illustrated in the indicated figures. nparLD, non-parametric, robust rank-based method for longitudinal (repeated measures) data analysis (Noguchi et al., 2012). K-W, non-parametric Kruskal-Wallis rank sum test.
